# Comparative evaluation of regression and machine-learning models for hepatocellular carcinoma risk stratification across diverse aetiologies

**DOI:** 10.1016/j.jhepr.2026.101740

**Published:** 2026-02-03

**Authors:** Pierre Nahon, Richard Layese, Pierre-André Natella, Lucia Parlati, Tounes Saidi, Nathalie Ganne-Carrié, Gisèle N’Kontchou, Cendrine Chaffaut, Jean-Charles Nault, Jessica Bamba-Funck, Angela Sutton, Clovis Lusivika Nzinga, Fabrice Carrat, Etienne Audureau

**Affiliations:** 1 AP-HP, Hôpitaux Universitaires Paris Seine Saint-Denis, APHP, Liver Unit, Université Sorbonne Paris Nord, F-93000 Bobigny, Inserm, UMR-1138 “Functional Genomics of Solid Tumors”, Centre de recherche des Cordeliers, Université de Paris, Paris, France; 2 Univ Paris Est Créteil, INSERM, IMRB, Equipe CEpiA (Clinical Epidemiology and Ageing), Unité de Recherche Clinique (URC Mondor), Service de Santé Publique, Assistance Publique Hôpitaux de Paris (AP-HP), Hôpitaux Universitaires Henri Mondor, F-94000, Créteil, France; 3 Université de Paris, département d’hépatologie/Addictologie, Hôpital Cochin, APHP, Paris, France; 4 Clinical Research Department, ANRS | Emerging Infectious Diseases, Paris, France; 5 SBIM, APHP, Hôpital Saint-Louis, Paris, Inserm, UMR-1153, ECSTRA Team, Paris, France; 6 Université Sorbonne Paris Nord, Laboratory for Vascular Translational Science, LVTS, INSERM, UMR 1148, F-93430, Villetaneuse, Université de Paris, F-75018, Paris, AP-HP, HUPSSD, Hôpital Avicenne, Laboratoire de Biochimie, F-93000, Bobigny, France; 7 Sorbonne Université, Inserm, Institut Pierre Louis d'Epidémiologie et de Santé Publique, AP-HP, Hôpital Saint-Antoine, Unité de Santé Publique, Paris, France

**Keywords:** cirrhosis, HCC risk stratification, machine learning, surveillance, cost-effectiveness

## Abstract

**Background & Aims:**

We aimed to develop machine learning (ML) models for hepatocellular carcinoma (HCC) risk stratification in patients with cirrhosis and to test their ability to identify those with an annual HCC incidence >3%, for whom more intensive surveillance may be justified.

**Methods:**

Data from three prospective cohorts (ANRS CO12 CirVir, CO22 Hepather, APHP CIRRAL) were analyzed. All patients underwent semiannual ultrasound surveillance and were randomly split into training and validation sets. HCC incidence was evaluated using a competing risk framework. A single tree (ST) model was developed using conditional decision trees, while random forest (RF) models were built by aggregating 1,000 trees. A deep neural network (DNN)–based survival model was also applied. ML model performance was compared with established regression-based scores: aMAP (age–male–ALBI–platelets) and FASTRAK (FAST-MRI for HCC suRveillance in pAtients with high risK of liver cancer).

**Results:**

Among 4,867 patients with non-viral cirrhosis or resolved/controlled viral cirrhosis, 294 (9.2%) developed HCC over a median follow-up of 61.5 months (annual incidence: 1.99%). The ST model identified four key predictors, generating five distinct risk groups. These included patients with mildly impaired liver function or those with elevated GGT and low platelet counts. The RF and DNN approaches confirmed ST findings and delineated complex interactions among predictors. Performance metrics (C-index, Brier score, decision curve analysis) showed no significant advantage of ML models over aMAP and FASTRAK. Calibration was consistent across models. ML models identified higher proportions of patients with an annual HCC incidence >3% (ST 44%; DNN 37%; RF 30%) compared with aMAP (36%) and FASTRAK (29%).

**Conclusions:**

ML-based algorithms did not outperform traditional risk scores but provided novel insights into variable interactions and helped identify clinically relevant patient subgroups with differing HCC risk profiles.

**Impact and implications:**

Accurate stratification of hepatocellular carcinoma risk in cirrhosis is essential to optimize surveillance strategies, and this study provides a scientific rationale for exploring machine learning approaches to capture complex, non-linear interactions among clinical variables beyond traditional regression models. Although machine learning did not improve predictive performance over established scores, it revealed clinically meaningful risk subgroups defined by liver function, platelet count, and GGT, underscoring its value as an interpretative and hypothesis-generating tool. These results are particularly relevant for hepatologists and clinical researchers seeking to refine risk-adapted surveillance and to inform the design of future models or trials.

## Highlights

• ML models were developed to stratify HCC risk in patients with cirrhosis.
• A simple decision tree identified four predictors and defined five clinically meaningful HCC risk groups.
• High-risk groups (annual HCC risk >3%) accounted for 44% of patients.
• Random forest and deep neuronal network approaches confirmed complex predictor interactions.
• ML models did not outperform clinical scores but improved clinical interpretability.

## Graphical abstract

**Figure:**
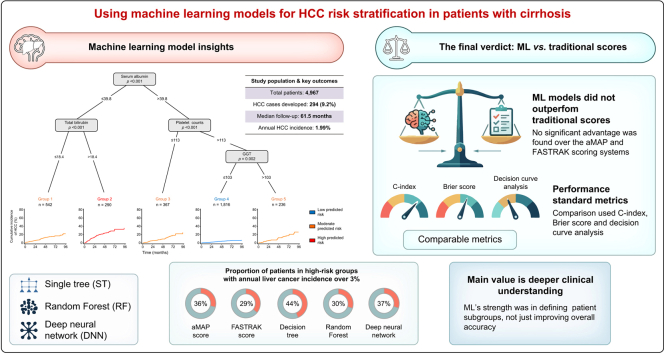

## Introduction

Early detection of hepatocellular carcinoma (HCC) through regular surveillance allows for the application of curative treatments, significantly improving outcomes for patients with cirrhosis or advanced chronic liver disease (ACLD).^1^ Although semiannual liver ultrasound remains the recommended primary imaging method due to its accessibility and safety, advanced diagnostics such as circulating biomarkers and specialized liver MRI protocols are under investigation for their enhanced diagnostic accuracy.^2^^,^^3^ However, these more costly and less widely available tools may only be cost-effective for patients with the highest risk of liver cancer, particularly those with an annual incidence exceeding 3%.^4^

The cost-effectiveness of surveillance strategies is largely influenced by HCC incidence, the aetiologies of which have shifted considerably over the past decade.^5^ Alcohol use and metabolic-associated steatotic liver disease (MASLD) have become the leading risk factors for cirrhosis and HCC,[6], [7], [8] while the prevalence of untreated HCV and HBV infections has significantly declined.^9^ These trends have led to a reduced overall risk of HCC among patients with cirrhosis compared to historical cohorts, with current annual incidence rates ranging from 1% to 2% in patients with compensated non-viral ACLD or controlled HBV/cured HCV infections.^8^^,^[10], [11], [12]

Several risk models and scoring systems have been designed to assess the likelihood of HCC development in patients with cirrhosis, typically integrating clinical, laboratory, and demographic factors to estimate individual risk.^3^^,^^13^ These scoring systems may in the near future assist clinicians in identifying individuals at higher risk of developing HCC, thus enabling targeted surveillance strategies and interventions for early detection that are currently being tested in randomized trials.^3^^,^^14^ More recently, machine learning (ML) approaches have shown promise in risk stratification by utilizing various clinical, laboratory, imaging, and genetic data to predict an individual's risk of developing a given disease.^15^ ML algorithms, including those based on decision trees, have the theoretical capability of detecting intricate interactions among predictive factors and thus help identifying more specific high-risk subgroups. By delivering more accurate personalized risk assessments, ML approaches have the potential to enhance HCC risk stratification, promote early detection, and improve patient outcomes.

This study aimed to develop ML models for HCC risk stratification in large prospective cohorts of patients with cirrhosis, taking into account recent changes in the ACLD epidemiological landscape, and to evaluate their relative performance against scoring systems based on conventional biostatistical approaches.

## Patients and methods

### Patients

The present work used data from one randomized clinical trial dedicated to HCC surveillance and three French prospective cohorts of adults with biopsy-proven compensated cirrhosis without detectable suspected focal liver lesions: the HCC 2000 trial,^16^ the ANRS CO12 CirVir cohort,^17^ the CIRRAL cohort,^12^ and the ANRS CO22 Hepather cohort.^18^ Each study was conducted in accordance with the ethical guidelines of the 1975 Declaration of Helsinki and French laws for biomedical research and was approved by Ethics Committees. All patients gave written informed consent to participate.

All patients enrolled in these cohorts underwent periodic liver ultrasound surveillance in accordance with international and French guidelines, with or without measurement of serum alpha-fetoprotein (AFP) levels. In cases where focal liver lesions were detected, a recall diagnostic work-up using contrast-enhanced imaging (CT or MRI) and/or image-guided biopsy was performed in accordance with the 2005 AASLD guidelines, updated in 2011.^19^^,^^20^ A diagnosis of HCC was thus established by either histological examination or based on probabilistic non-invasive criteria (mainly dynamic imaging revealing early arterial hyperenhancement and washout on portal venous or delayed phases) according to the different time periods (before and after 2011). When HCC diagnosis was established, treatment was determined using a multidisciplinary approach according to AASLD^19^^,^^20^ and the EASL–EORTC^21^ guidelines.

In addition to HCC occurrence, which was the primary endpoint of all four cohorts, all events that occurred during follow-up (*i.e*. death, liver decompensation,^22^ bacterial infection,^23^ extrahepatic malignancies^24^ and cardiovascular diseases^25^) were recorded using information obtained from the medical records of patients held by each centre. Moreover, likely cause(s) of death were established. Patients who underwent liver transplantation were censored for analysis at the date of transplantation. All treatments, including antiviral therapies, were recorded at inclusion, and patients were notified of any modifications during follow-up. A single database encompassing clinical data from the four cohorts was built on November 18, 2019.^26^ Among all included patients, only those with non-viral causes of cirrhosis (alcohol- and/or MASLD-related) or those who achieved HBV control/HCV eradication during follow-up were considered for the present analyses.

#### ANRS CO12 CirVir cohort

The ANRS CO12 CirVir cohort, sponsored and funded by the ANRS (France REcherche Nord & Sud Sida-HIV Hépatites), is a multicentre observational cohort that aims to characterize the incidence of complications occurring in biopsy-proven compensated cirrhosis and to identify the associated risk factors using competing risks analysis.^17^ The full CirVir protocol is available via the ANRS Web site (http://anrs.fr). Specific additional inclusion criteria were i) cause of cirrhosis related to either chronic infection with HCV and/or HBV regardless of the levels of replication and alcohol consumption, ii) patients belonging to Child-Pugh A at enrolment, iii) absence of previous hepatic complications (particularly ascites, gastrointestinal haemorrhage, or HCC), and iv) absence of severe uncontrolled extrahepatic disease resulting in an estimated life expectancy of less than 1 year.

Among 1,822 patients recruited in 35 French clinical centres between March 2006 and July 2012, 151 were subsequently excluded from analysis after reviewing individual data due to either non-compliance with inclusion criteria (n = 142) or consent withdrawal (n = 9), leading to a total of 1,671 patients selected for further analysis, including the present study.

#### CIRRAL cohort

CIRRAL is a multicentre cohort study implemented in 22 French and 2 Belgian tertiary liver centres to capture the whole spectrum of complications occurring in compensated alcohol-related cirrhosis (complicated or not by metabolic syndrome) using competing risk analyses.^12^ The promoter was the APHP. The cohort was funded by the French National Institute of Cancer (INCa), the French Association for Research in Cancer and the ANRS (PAIR CHC 2009) and was registered on ClinicalTrials.gov (NCT00190385). Specific additional inclusion criteria were i) cause of cirrhosis related to chronic alcohol abuse according to the World Health Organization criteria (more than 21 glasses per week for females and more than 28 glasses per week for males) for at least 10 years, ii) absence of chronic infection with HCV or HBV, and iii) patients belonging to Child-Pugh A at enrolment. The follow-up of patients was strictly superposed to the ANRSCO12Cirvir cohort design.

Among 706 patients included between October 2010 and April 2016, 54 were subsequently excluded after reviewing individual data because of violations of the inclusion criteria (n = 48) or consent withdrawal (n = 6); ultimately, a total of 652 patients were selected for further analysis, including the present study.

#### ANRS CO22 Hepather cohort

The ANRS CO22 Hepather cohort is a French national, multicentre, prospective, observational cohort study of patients with HBV or HCV infection that started in August 2012, among whom 3,045 had active HCV-related cirrhosis at inclusion.^18^ Among the latter, a subset of 1,374 patients consecutively enrolled between 08/2012 and 01/2014 who responded to similar inclusion criteria as those included in the CirVir and CIRRAL cohorts were selected. Follow-up, antiviral treatments, and the definition of the endpoint were identical to those in the CirVir cohort.

### Endpoint

HCC cumulative incidence was the primary endpoint for predictive modelling. The baseline was defined as the date of inclusion in the corresponding cohort for patients with non-viral causes of cirrhosis and the date of sustained virological response achievement/viral control for patients with HCV/HBV-related cirrhosis. All analyses were conducted under a competing risk framework, considering death as a competing event for HCC occurrence.

### Statistical analyses

#### Descriptive statistics

Descriptive results are presented as medians (IQR) for continuous variables and as numbers (percentages) for categorical data. Comparison of characteristics between groups were performed using the Mann-Whitney rank-sum test for continuous variables and the chi-squared test or Fisher’s exact test for categorical variables.

### Predictive modelling

To fulfil the primary objective of this analysis, different modelling approaches for HCC risk stratification were developed and validated using the three aforementioned cohorts. To ensure appropriate separation between development and validation data, each of the three cohorts was split into a training set (two thirds of patients) and a validation set (one third) using center-based random sampling. Entire centers were randomly assigned to either the training or the validation dataset so that no center contributed patients to both sets. Following this center-level split, missing data in the training and validation sets were imputed independently using the missForest algorithm to avoid any risk of information leakage. Fig. S1 describes the methodology used for the constitution of training and validation sets.

Several modeling approaches to HCC prediction were implemented and compared for their predictive performance and clinical significance. First, decision tree-based ML methods were applied. The models included previously identified and validated risk factors for HCC, commonly used in traditional risk scoring systems based on multivariate Cox proportional hazards modeling. These variables included age, sex, markers of liver condition (serum aspartate aminotransferase, alanine aminotransferase, gamma-glutamyltransferase [GGT], AFP), and surrogate indicators of portal hypertension (platelet count) or liver failure (prothrombin time, total and direct bilirubin levels). In addition to these established HCC risk factors, the ML models also incorporated routine biological parameters that were consistently available in all patient medical records: international normalized ratio, serum creatinine, blood glucose (glycemia), alkaline phosphatase, and serum ferritin.

For illustrative purposes, a single decision tree (ST) was initially built by recursive partitioning analysis using the conditional inference tree methodology,^27^ because of its visual appeal to display the main relationships at play. Starting with all observations, the algorithm automatically identifies the optimal splits in data to partition the population into subgroups with differentiated HCC risks, repeating the process recursively until a stopping criterion is met.

Because of the ST approach’s tendency for overfitting the training data, we then derived prognostic algorithms using a random (survival) forest (RF) approach for censored data, taking into account a competing risks framework. RFs combine the results obtained from a large ensemble of trees, thus avoiding the problem of selecting a single tree of appropriate size and often producing more stable predictive models.^28^ In the present analysis, we used 1,000 trees, selected based on an examination of the out-of-bag error rate across a range of 100–3,000 trees. The out-of-bag error rate stabilized around 1,000 trees, with no meaningful improvement in model performance beyond this point (see Fig. S2). Unlike Fine-Gray modeling or single decision trees, RFs do not produce regression coefficients or decision paths to enable direct interpretation of the complex underlying prognostic model, so they are sometimes viewed as “black-boxes”. Variable importance measures were thus computed to help quantify the importance of each predictor within the RF, by examining the increase in prediction error when a perturbation is added to the variable. To optimize the RF model, we used the mlr3 R package to select the most important features to keep in the model^29^ and to perform hyperparameter tuning using a grid search with internal five-fold cross-validation on the development set. Optimal values for the mtry (range 2–50) and node size (range 2–50) parameters – corresponding to the number of variables sampled at each split and the degree of pruning in each decision tree, respectively – were identified.

Finally, we implemented a deep neural network (DNN)-based survival model to estimate survival probabilities without relying on the proportional hazards assumption, using the DeepHit framework.^30^ DeepHit can accommodate non-linear and potentially non-proportional relationships between covariates and event times and explicitly models competing risks by predicting the joint distribution of survival times and event types, thereby offering greater flexibility. Hyperparameters were optimized using Optuna, a Bayesian optimization framework.

The performances of the machine learning models were compared to those of two previously published HCC risk scores, both derived using multivariate Cox proportional hazards modeling or Fine-Gray modeling and developed in contemporary cohorts of patients with ACLD without active viral replication.

The first was the age–male–ALBI–platelets (aMAP) score,^31^ which includes age, sex, ALBI score, and platelet count. The second was the FASTRAK score, previously developed by our group in a subset of the present population under study,^4^ and currently used in an ongoing randomized controlled trial^14^ investigating the addition of FAST-MRI in high-risk patients with cirrhosis, defined by an annual HCC incidence >3% (FASTRAK trial, NCT05095714; FAST-MRI for HCC suRveillance in pAtients with high risK of liver cancer). This score incorporates age, sex, platelet count, GGT, total bilirubin, and AFP.

For all modeling approaches, risk classes were generated based on quintiles of the predicted 3-year HCC risk score. The discriminative performance of the prognostic models was assessed in the validation set using time-dependent AUC^32^ and Brier score, which measure the probability of concordance between predicted and observed survival,^33^ along with their confidence intervals. We also estimated the standardized ‘net benefit’ derived from decision curve analysis^34^^,^^35^), a method that is increasingly used for evaluating alternative diagnostic or prognostic strategies, helping to identify the one with the highest clinical utility or ‘net benefit’. Finally, calibration plots were generated to assess the agreement between observed outcomes and predicted survival probabilities. Survival curves were plotted using the estimated cumulative incidence function from the competing risks framework. Finally, for each model we calculated the proportion of patients with an annual HCC incidence >3%.

Except for descriptive analyses, all other analyses were conducted on imputed data, with imputation performed using a RF-based approach^36^ with the R package *missForest*. Finally, as all newly developed models were based on ML approaches (including RFs), explicit feature decorrelation was not performed, as these algorithms are generally robust to correlated predictors.

Statistical analyses were performed using Stata v17.0 (StataCorp, TX, USA), R v4.4.1 (R Foundation for Statistical Computing, Vienna, Austria; using party, partykit,^27^ mlr3, missForest, riskRegression and pec packages) and Python 3.7 (DeepHit library)

## Results

### Selection and baseline characteristics of patients

A total of 5,624 patients with compensated cirrhosis undergoing HCC surveillance and included in the three cohorts were considered (see flowchart, Fig. S3). Among them, 757 were excluded, mostly because of persistent HCV/HBV viral infection during follow-up. The remaining 4,867 patients had either non-viral causes of cirrhosis and/or cured HCV/controlled HBV infections and were included in all subsequent analyses. Their baseline characteristics are displayed in Table 1 as a function of their inclusion in the development (n = 3,251) or validation (n = 1,616) sets. Table S1 also provides the baseline characteristics of patients as a function of their inclusion in the three cohorts.

**Table 1:** Baseline characteristics of the studied population.

	Available data, n	Development cohort n = 3,251	Validation cohort n = 1,616	ASMD∗	*p* value
Age	4,867	59.1 ± 10.7 58.7 [52.0–66.0]	58.8 ± 10.9 58.0 [51.7–66.0]	0.028	0.35
Male sex	4,867	2196 (67.5)	1084 (67.1)	0.010	0.75
Platelet count, 10^3^/mm^3^	4,223	161 [115–210]	159 [109–205]	0.032	0.26
AST	4,259	29.0 [23.0–39.0]	29.0 [23.0–39.0]	0.014	0.93
ALT	4,397	26.0 [19.0–38.0]	26.0 [19.0–38.0]	0.009	0.43
GGT	4,082	45.0 [26.0–91.0]	45.0 [27.8–95.0]	0.055	0.14
Prothrombin time, %	2,955	87.0 [76.0–97.0]	86.0 [76.0–95.0]	0.120	**0.007**
Serum albumin, G/L	3,352	42.0 [38.8–45.0]	42.0 [39.0–45.0]	0.043	0.79
Total bilirubin, μmol/L	3,578	11.0 [8.0–16.0]	11.0 [8.0–16.2]	0.010	0.70
Alpha-fetoprotein, ng/ml	3,842	5.1 [3.00–10.5]	5.2 [3.00–10.2]	0.009	0.84
INR	2,685	1.10 [1.01–1.20]	1.10 [1.05–1.19]	0.025	0.15
Creatinine	3,139	71.0 [61.3–83.1]	71.6 [61.9–84.6]	0.037	0.25
Glycemia	2,851	1.03 [0.90–1.60]	1.04 [0.90–1.64]	0.008	0.46
Alkaline phosphatase	4,019	89.0 [69.0–124]	86.5 [67.0–114]	0.118	**0.002**
Ferritin	4,867	239 [107–491]	235 [100–502]	0.036	0.75
Cirrhosis aetiology	4,867				0.065
Cured HCV		2,377 (73.1%)	1,166 (72.2%)	0.022	
Controlled HBV		464 (14.3%)	210 (13.0%)	0.007	
Alcohol and/or metabolic		410 (12.6%)	240 (14.9%)	0.041	
Cohorts	4,867				0.094
CIRRAL		410 (12.6%)	240 (14.9%)	0.065	
CirVir		657 (20.2%)	322 (19.9%)	0.037	
HEPATHER		2,184 (67.2%)	1,054 (65.2%)	0.065	

ALT, alanine aminotransferase; ASMD, absolute standardized mean difference; AST, aspartate aminotransferase; GGT, gamma-glutamyltransferase; INR, international normalized ratio.

Results are given as n (%) for categorical variables and mean (±SD) or median (IQR) for continuous variables.

∗ A value of ASMD >0.10 denotes an imbalance of the studied parameter between the two groups.

### HCC incidence and survival rates

After a median follow-up of 59.3 (95% CI 57.8; 60.5) months in the development set, 299 (9.2%) patients developed HCC, with a corresponding yearly incidence of 2.06% (95% CI 1.84–2.31). Similarly, 158 (9.8%) HCC cases occurred after 66.5 (95% CI 65.0–67.8) months in the validation set (annual incidence: 2.00% (95% CI 1.72–2.35). The HCC incidence was similar in both sets (subdistribution hazard ratio 0.99, 95% CI 0.82; 1.21, *p =* 0.915, Fig. S4). During the same timeframe, 396 (12.2%) patients died in the development set. In the validation set, 197 (12.2%) patients died. Overall survival was also similar in both populations (hazard ratio 0.91, 95% CI 0.76–1.07, *p =* 0.254).

### Prognostic model using single decision tree recursive partitioning

**Fig. 1:**
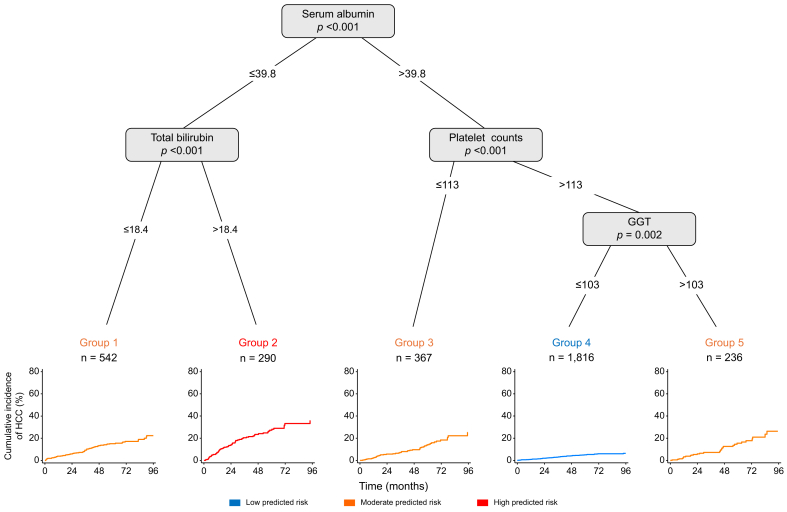
Decision tree from recursive partitioning analysis of time to HCC occurrence (development cohort; n = 3,251 patients, of whom 299 developed an HCC). Low-predicted HCC risk groups had an annual HCC incidence <1% (n = 1,816, 56%, annual incidence 0.9%); high-predicted HCC risk groups had an annual HCC incidence >5% (n = 290, 9%, annual incidence 6.6%); intermediate-predicted HCC risk groups had an annual HCC incidence between 1% and 5% (n = 1,145, 35%, annual incidence 3.6%). GGT, gamma-glutamyltransferase; HCC, hepatocellular carcinoma.

For illustrative purposes and to aid in understanding the main interactions between predictors, a recursive partitioning approach was used to build the single decision tree shown in Fig. 1.

Four main predictors were identified by the algorithm, yielding five groups from various combinations of these predictors and demonstrating markedly contrasting risks of HCC, as shown by the corresponding curves at each end node. Low-predicted HCC risk groups had an annual HCC incidence <1% (n = 1,816, 56%, annual incidence 0.9%); high-predicted HCC risk groups had an annual HCC incidence >5% (n = 290, 9%, annual incidence 6.6%); intermediate-predicted HCC risk groups had an annual HCC incidence between 1% and 5% (n = 1,145, 35%, annual incidence 3.6%).

The most predictive factor in the root node was serum albumin which dichotomized patients into two main subpopulations. Although all patients had Child-Pugh A cirrhosis at inclusion, a slightly impaired albumin level below 39.8 G/L selected patients with a high HCC risk profile, who were furthermore stratified as moderate-(Group 1) or high- (Group 2) risk groups as a function of bilirubin level, the latter group representing an “extreme” phenotype comprising a small subpopulation (n = 290, 9% of the population). Among patients with higher serum albumin levels, surrogate markers of portal hypertension (platelet count) or comorbidities/liver insult (GGT levels) defined several clusters of patients. In particular, patients with high platelet counts and low GGT levels comprised a large group of 1,816 patients (56%, Group 4) with a particularly low HCC incidence. By contrast, patients with either a low platelet count (<113 x10^3^/mm^3^) or high GGT levels (>103 IU/L) identified patients with a moderate risk (Groups 3 and 5).

The clinical and biological characteristics of the five groups generated by decision tree analysis are shown in Table 2. In Group 2, the high-risk group, several additional features were impaired beyond the criteria identified by the decision tree (*e.g*. higher alkaline phosphatase levels and lower prothrombin time) and this group included a higher proportion of patients with metabolic dysfunction- and alcohol-associated liver disease. By contrast, patients in Group 4, representing the lowest HCC risk, were characterized by generally optimal liver parameters and minimal indirect signs of comorbidities, and were enriched for patients with cured or controlled viral-induced cirrhosis.

**Table 2:** Comparison of the features of the final groups obtained by decision tree analysis (development cohort; n = 3,210 patients, of whom 294 developed an HCC).

	Group 1	Group 2	Group 3	Group 4	Group 5	*p* value
	n = 542	n = 290	n = 367	n = 1,816	n = 236	
Age	60.9 [54.0–68.9]	59.0 [52.9–67.0]	58.0 [51.8–65.7]	58.0 [51.9–65.8]	57.0 [50.0–63.7]	**<0.001**
Male sex	340 (62.7)	194 (66.9)	233 (63.5)	1,258 (69.3)	171 (72.5)	**0.009**
Platelet count, 10^3^/mm^3^	146 [101–197]	94.0 [66.0–135]	89.0 [71.0–102]	186 [156–217]	168 [141–206]	**<0.001**
AST	31.1 [24.0–43.6]	45.0 [34.0–60.0]	32.0 [26.0–44.0]	25.0 [21.0–30.0]	40.5 [30.5–59.5]	**<0.001**
ALT	26.0 [18.0–35.0]	29.0 [22.0–40.0]	29.0 [22.0–43.0]	24.0 [19.0–32.0]	43.0 [29.0–59.0]	**<0.001**
GGT	62.0 [34.0–122]	86.1 [46.0–156]	57.0 [33.0–113]	31.0 [22.0–46.0]	165 [130–259]	**<0.001**
Prothrombin time,	80.0 [72.0–93.0]	68.0 [58.7–75.9]	81.0 [73.0–90.0]	92.6 [86.0–97.3]	88.8 [80.5–96.0]	**<0.001**
Serum albumin, G/L	37.9 [36.0–39.0]	35.4 [32.8–37.5]	42.7 [41.0–44.6]	43.9 [42.3–45.1]	43.1 [41.6–45.0]	**<0.001**
Total bilirubin, μmol/L	10.3 [8.0–14.0]	26.0 [21.7–34.0]	12.5 [9.7–17.0]	9.8 [8.0–11.8]	10.5 [8.0–14.0]	**<0.001**
Alpha-fetoprotein, ng/ml	5.6 [3.50–10.3]	6.3 [4.00–9.8]	6.7 [3.80–11.8]	5.0 [3.00–9.3]	6.5 [4.00–11.6]	**<0.001**
INR	1.17 [1.09–1.25]	1.30 [1.20–1.46]	1.14 [1.10–1.22]	1.07 [1.02–1.12]	1.10 [1.04–1.16]	**<0.001**
Creatinine	70.6 [62.2–83.0]	64.2 [55.1–74.9]	68.7 [60.5–78.8]	72.8 [65.5–81.3]	71.3 [62.7–77.9]	**<0.001**
Glycemia	1.19 [1.01–1.91]	1.31 [1.11–5.1]	1.08 [0.92–1.30]	1.00 [0.93–1.10]	1.19 [1.04–4.00]	**<0.001**
Alkaline phosphatase	103 [81.0–129]	126 [97.0–154]	99.0 [79.1–121]	82.0 [66.0–103]	109 [84.3–140]	**<0.001**
Ferritin	153 [78.0–344]	163 [80.0–358]	225 [125–425]	254 [149–441]	280 [161–548]	**<0.001**
Cirrhosis aetiology						**<0.001**
Cured HCV	367 (67.7)	173 (59.7)	266 (72.5)	1,415 (77.9)	156 (66.1)	
Controlled HBV	60 (11.1)	26 (9.0)	47 (12.8)	311 (17.1)	20 (8.5)	
Alcohol and/or metabolic	115 (21.2)	91 (31.4)	54 (14.7)	90 (5.0)	60 (25.4)	
Cohorts						**<0.001**
CIRRAL	115 (21.2)	91 (31.4)	54 (14.7)	90 (5.0)	60 (25.4)	
CirVir	91 (16.8)	33 (11.4)	93 (25.3)	396 (21.8)	44 (18.6)	
HEPATHER	336 (62.0)	166 (57.2)	220 (59.9)	1,330 (73.2)	132 (55.9)	

### Prognostic models using random survival forests

**Fig. 2:**
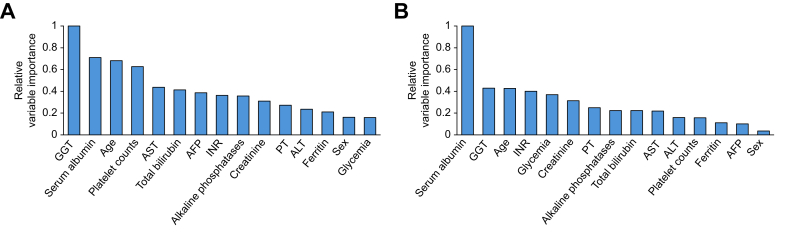
Variable importance from random survival forest analysis. Variable importance from random survival forest analysis of time to HCC occurrence (A) and HCC-free mortality (B) in the development cohort (n = 3,251 patients, of whom 299 developed an HCC). AFP, alpha-fetoprotein; ALT, alanine aminotransferase; AST, aspartate aminotransferase; GGT, gamma-glutamyltransferase; HCC, hepatocellular carcinoma; PT, prothrombin time.

Random forests were constructed by aggregating 1,000 decision trees (Fig. S1). Fig. 2 ranks the predictors in order of their relative importance in the RF algorithm, with high importance values indicating the most influential variables predictive of HCC (Fig. 2A) or death without HCC (Fig. 2B). RF approaches confirmed the results of the single decision tree analysis previously described, identifying higher GGT, lower platelet count and albumin levels, but also older age, higher AST and AFP levels as predictive of HCC. Similar to the decision tree analysis, other variables had a weaker influence.

### Prognostic models using deep neural networks

We performed five-fold cross-validation on the training set to identify the optimal hyperparameters, selecting those that maximized the average time-dependent AUC (calculated as the mean of three AUCs at 12, 36, and 60 months). The resulting DNN consisted of a shared hidden layer (300 neurons, ELU activation) followed by three cause-specific subnetworks, each with three hidden layers of 300 neurons and ELU activation. The network was trained for 1,000 iterations with a batch size of 128, 40% dropout, and optimized regularization parameters (α = 0.1, β = 3, γ = 0.5). These optimized hyperparameters were then applied to evaluate model performance in the validation cohort (see Table 3 and S2).

### Discriminative performance and calibration in the validation cohort

**Fig. 3:**
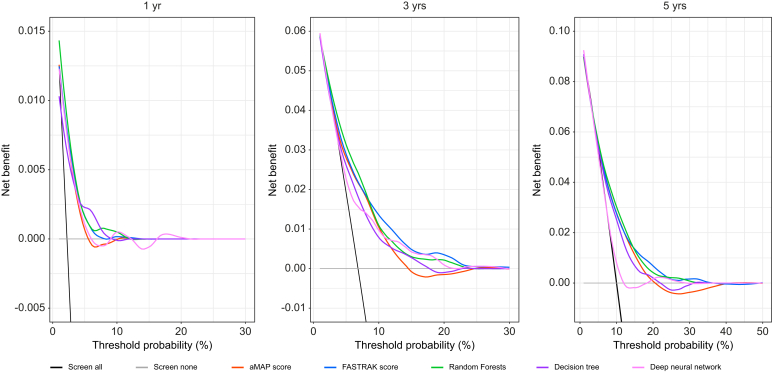
Decision curves display the net benefit between surveillance benefits and surveillance harms. Net benefit in screened patients is calculated across a range of HCC risk thresholds (defined as the minimum probability of disease at which biannual screening would be warranted), as the proportion of patients with true positive results minus the proportion with false-positives multiplied by the odds at the threshold probability (HCC risk/1 – HCC risk). The net benefits of the risk prediction models were compared with those from two different reference strategies of screening all patients or none. Risk models with higher discriminative power will provide higher net benefit, as evidenced by the highest plotted decision curves. HCC, hepatocellular carcinoma.

The discriminative performance of the aMAP and FASTRAK scores, as well as RF and DNN models, at 1, 3 and 5 years was computed and compared in the validation cohort using time-dependent C-indices and Brier scores (Table 3). Comparisons of these metrics did not indicate any substantial advantage of the ML models over traditional scores for HCC prediction at 1-, 3-, or 5-year horizons, a finding further confirmed by decision curve analysis (Fig. 3). Table S2 additionally shows the performances of the different models applied in each of the three cohorts, yielding similar conclusions (Table S2).

**Table 3:** Discrimination performance indices by modeling approach.

	Training set	Validation set
**Time-dependent AUC**		
At 1 year		
aMAP score	0.7380 (0.6558. 0.7921)	0.7285 (0.6488–0.8082)
FASTRAK score	0.7846 (0.7207–0.8265)	0.7207 (0.6385–0.8030)
Decision tree	0.6620 (0.5686. 0.7560)	0.6778 (0.5888–0.7667)
Survival random forest	0.7480 (0.6920–0.8123)	0.7441 (0.6683–0.8199)
Deep neural network	0.7913 (0.7388–0.8437)	0.7525 (0.6731–0.8320)
At 3 years		
aMAP score	0.7491 (0.7133–0.7918)	0.6953 (0.6400–0.7506)
FASTRAK score	0.7464 (0.6931–0.7956)	0.7154 (0.6630–0.7678)
Decision tree	0.6746 (0.6211–0.7311)	0.6682 (0.6143–0.7220)
Survival random forest	0.7364 (0.6832–0.7851)	0.7263 (0.6778–0.7749)
Deep neural network	0.7851 (0.7526–0.8175)	0.7245 (0.6727–0.7764)
At 5 years		
aMAP score	0.7021 (0.6599–0.7322)	0.6755 (0.6273–0.7237)
FASTRAK score	0.7062 (0.6585–0.7400)	0.6893 (0.6418–0.7368)
Decision tree	0.6501 (0.5792–0.6988)	0.6499 (0.6029–0.6969)
Survival random forest	0.7040 (0.6598–0.7481)	0.6832 (0.6349–0.7315)
Deep neural network	0.7369 (0.7052–0.7686)	0.6986 (0.6496–0.7476)

**Brier score**		
At 1 year		
aMAP score	0.022 (0.015–0.030)	0.021 (0.015–0.028)
FASTRAK score	0.022 (0.015–0.030)	0.021 (0.015–0.028)
Decision tree	0.022 (0.015–0.030)	0.021 (0.015–0.028)
Survival random forest	0.022 (0.014–0.030)	0.021 (0.015–0.028)
Deep neural network	0.021 (0.016–0.025)	0.022 (0.015–0.028)
At 3 years		
aMAP score	0.055 (0.050–0.065)	0.062 (0.051–0.073)
FASTRAK score	0.055 (0.050–0.064)	0.060 (0.050–0.071)
Decision tree	0.058 (0.052–0.068)	0.062 (0.051–0.073)
Survival random forest	0.055 (0.050–0.064)	0.061 (0.051–0.072)
Deep neural network	0.055 (0.048–0.061)	0.062 (0.052–0.071)
At 5 years		
aMAP score	0.086 (0.079–0.098)	0.087 (0.075–0.100)
FASTRAK score	0.085 (0.079–0.096)	0.086 (0.074–0.098)
Decision tree	0.090 (0.083–0.101)	0.087 (0.075–0.099)
Survival random forest	0.085 (0.079–0.097)	0.087 (0.075–0.099)
Deep neural network	0.087 (0.080–0.094)	0.090 (0.079–0.100)

**Fig. 4:**
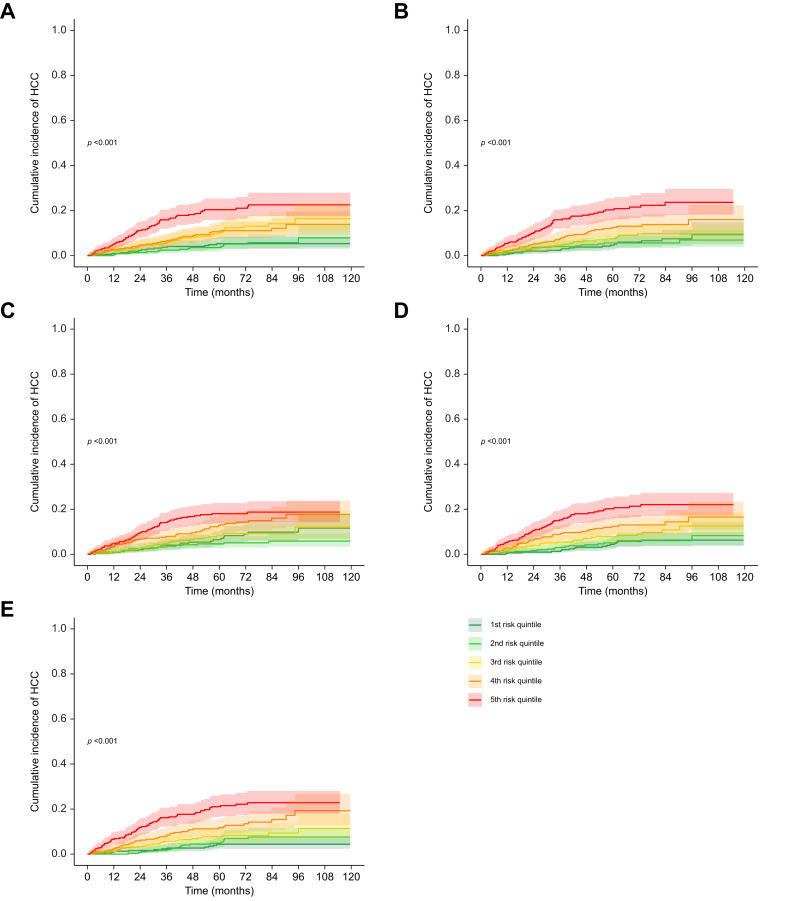
Cumulative incidence curves of HCC by 3-year quintiles of predicted risk according to different modelling approaches using the whole follow-up of the validation cohort (n = 1,616 patients, of whom 158 developed an HCC). (A) aMAP score. (B) FASTRAK score. (C) Decision tree. (D) Random survival forest. (E) Deep neural network. Levels of significance: *p* <0.05) (Gray's test). HCC, hepatocellular carcinoma.

**Fig. 5:**
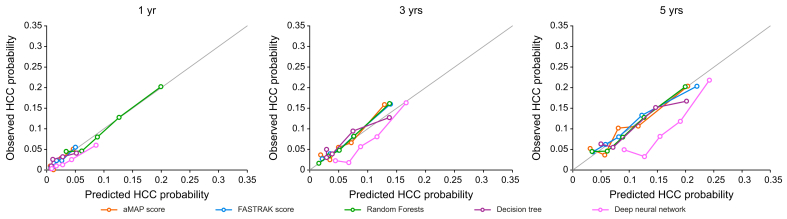
Calibration plots of machine learning and traditional models in the validation population (n = 1,616 patients, of whom 158 developed an HCC). HCC, hepatocellular carcinoma.

To illustrate the clinical value of ML models in discriminating patients with different risk levels, five risk categories were defined based on quintiles of the predicted 3-year HCC risk score from each model. Fig. 4 shows the resulting incidence curves for the cumulative incidence of HCC according to aMAP/FASTRAK scores, decision tree, RF and DNN modeling approaches. Consistent with the computed C-indices and Brier scores, a clearly graded relation between predicted risk and observed HCC occurrence was apparent for both Cox models (aMAP/FASTRAK scores) and RF or DNN. Finally, calibration curves for the validation set showed that both ML approaches and traditional models maintained similar alignment between predicted and observed risks (Fig. 5).

Finally, for each model, we calculated the proportion of patients with an annual HCC incidence >3%. ML models identified higher proportions of patients with an annual HCC incidence >3%: ST 1,435/3,251 (44%); DNN 1,202/3,251 (37%); RF 975/3,251 (30%) *vs*. aMAP 1,179/3,251 (36%); FASTRAK 941/3,251 (29%).

## Discussion

The findings from the present report provide a comprehensive evaluation of both traditional and ML-based approaches in the context of HCC surveillance programmes. Our findings offer valuable insights into the use of ML methodologies for HCC risk prediction, demonstrating the potential and limitations of such models within clinical practice. Importantly, although the RF models revealed interactions between variables and identified patient clusters with differing risk levels – features not captured by traditional scoring systems – the overall predictive performance was comparable.

One of the key contributions of this study is the ML models’ ability to stratify patients into specific risk subpopulations and highlight individuals with particularly high-risk profiles. The identification of clinical predictors such as GGT levels, platelet counts, and albumin through the ST (Fig. 1) and RF (Fig. 2) models echoes known risk factors, suggesting that ML models can validate and potentially reinforce the clinical importance of variables well-documented in HCC risk literature.^37^ Algorithms using ML approaches applied to the French cohorts also provided additional insights compared with traditional logistic regression models. First, by demonstrating the interactions between variables, they help identify clusters of patients defined by different HCC risk levels. For instance, the recursive partitioning approach (Fig. 1, Table 2) shows that in this population, slightly decreased albumin levels in otherwise compensated patients, combined with higher bilirubin, define a small subset of patients (9%) with an annual HCC incidence >5%. This observation suggests that even minimal impairment of hepatic function has a major impact on the risk of HCC. Moreover, higher GGT levels – likely indicative of associated comorbidities – appear to further refine individual risk, particularly by identifying patients with otherwise perfectly compensated liver function who have an intermediate HCC risk of >3%. Beyond the notably illustrative aspect of these ST analyses for clinicians, the RF model, constructed using an ensemble of 1,000 decision trees, corroborated the predictors identified by the ST model and reinforced their importance (Fig. 2). Interestingly, epidemiological factors such as older age and non-specific biomarkers like higher GGT levels were among the most influential predictors, alongside parameters estimating liver function, whereas non-hepatic markers appeared to be less influential. Finally, the RF model also highlighted the complex interactions among variables, a strength of ML methods that may be overlooked in traditional regression-based approaches.

These observations could be pivotal for personalized clinical decision-making, allowing targeted resource allocation and enhanced monitoring strategies for patients identified as being at higher HCC risk. For instance, it has been shown that HCC surveillance based on liver MRI examination would be cost-effective for very-early HCC (BCLC 0) detection as soon as annual HCC incidence surpasses 3%.^4^ This hypothesis is currently being tested in the setting of a French randomized trial (FASTRAK trial, NCT NCT05095714), in which the selection of patients is based on the FASTRAK scoring system used as comparator in the present work, which has been shown to enable the identification of roughly 30% of patients with an annual HCC incidence >3%.^14^ This finding was confirmed in the present analyses conducted in larger cohorts. For instance, the present ST model showed that this proportion could be increased to 44% (intermediate- and high-predicted HCC risk groups, Fig. 1). The application of such a scoring system for allocation into MRI surveillance programmes would increase the detection of early-stage tumours eligible for curative treatments – such as surgery, ablation, or transplantation – a strategy highlighted by Europe’s Beating Cancer Plan as the most effective measure to improve the poor prognosis of liver cancer.^38^^,^^39^

Despite their added sophistication, ML models offered no substantial discriminative advantage over established scoring systems (Table 3 and S2, Fig. 4), even when flexible and highly complex approaches, such as DNNs, were included.

The extent to which ML models may help in detecting subtle patterns that emerge over longer follow-up periods remains to be explored. Calibration curves for the validation set showed that both ML approaches and traditional models maintained similar alignment between predicted and observed risks (Fig. 5).

Of note, our previously published FASTRAK score, initially developed in a subgroup of the present population,^4^ was validated following the incorporation of 1,864 (38%) additional patients. This suggests that while ML techniques can enrich data-driven risk stratification, their incremental benefit over conventional statistical methods may be context-dependent. The results indicate that existing models, such as the previously published aMAP^31^ or FASTRAK scores,^4^ are robust for immediate- and medium-term HCC risk prediction, while ML models might be better suited for scenarios in which predictive risk factors or biomarkers only relevant in highly specific subgroups can be identified. For example, genetic variants (*PNPLA3, TM6SF2*, and others) associated with HCC identified through large-scale pangenomic studies have shown only modest improvements in risk stratification when integrated into regression models in longitudinal cohorts under surveillance.^40^ These findings suggest that such genetic biomarkers may have a significant impact only in specific patient subgroups, which are not adequately captured by traditional statistical methods. The potential of newly identified risk factors to enhance HCC risk stratification using ML approaches is highly anticipated.^13^

Despite the promise of ML models, there are several considerations to note. The computational complexity and interpretability of ML models, especially when compared to simpler scoring systems, may pose challenges in clinical adoption. Traditional models benefit from their ease of use and clear clinical interpretation, which remain critical for broad implementation in clinical practice. In this context, the added complexity might not justify widespread substitution of established models without further validation across diverse populations and settings, particularly outside France. However, the ML models’ potential to uncover nuanced interactions might serve as a complementary tool, particularly in the presence of heterogeneous patient populations. It should also be noted that our analysis was limited to a selected set of ML approaches adapted for competing-risks survival analysis, and that other existing or future methods may yield improved performance. Finally, our findings are specific to the present dataset and the predictors available, and may not be generalizable to other populations or clinical settings. Future research should explore the integration of ML-based risk stratification tools into clinical workflows, potentially as complementary tools to traditional scores rather than replacements.

In conclusion, our study highlights that ML models can reveal interactions between variables, identify patient clusters with distinct risk profiles, and detect extreme phenotypes associated with particularly high HCC risk. However, when only combining clinical and biological routine parameters, they do not yet demonstrate a significant performance advantage over traditional models. Their enrichment by newly identified risk factors derived from circulating biobanks or imaging examinations is highly anticipated.^40^^,^^41^ The development and application of these models will need to balance predictive power, clinical utility, and interpretability to optimize patient care in HCC surveillance programmes.

## Abbreviations

AFP, alpha-fetoprotein; AST, aspartate aminotransferase; DNN, deep neuronal network; GGT, gamma-glutamyltransferase; HCC, hepatocellular carcinoma; ML, machine learning; ST, single tree; RF, random (survival) forest.

## Authors’ contributions

Drs Nahon and Audureau had full access to all data in the study and take responsibility for data integrity and the accuracy of data analysis. *Study concept and design:* Nahon, Audureau. *Acquisition of data:* Nahon, Parlati, Lusivika Nzinga, Carrat, Ganne-Carrié, N’Kontchou, Chaffaut, Bamba-Funck, Sutton. *Analysis and interpretation of data:* Nahon, Layese, Natella, Audureau. *Drafting of the manuscript:* Nahon, Layese, Audureau. *Critical revision of the manuscript for important intellectual content:* all authors. *Statistical analyses:* Layese, Audureau. *Obtained* funding*:* Nahon, Ganne-Carrié, Parlati, Carrat. *Administrative, technical and material* support*:* Nahon, Saidi, Layese, Natella, Audureau. *Study supervision:* Nahon, Audureau.

## Data availability

Data can be made available upon reasonable request.

## Declaration of generative AI and AI-assisted technologies in the writing process

During the preparation of this work the author(s) used ChatGPT in order to check for grammar/spelling. ChatGPT was also partially used to design the Graphical abstract. After using this tool, the authors reviewed and edited the content as needed and take full responsibility for the content of the publication.

## Financial support

The promoters of the three prospective cohorts were the Assistance Publique des Hôpitaux de Paris (APHP) for CIRRAL and ANRS for CirVir and Hepather. The cohorts were funded by 1) 1) the National Agency for Research on HIV and Hepatitis (10.13039/501100003323ANRS) for CirVir and Hepather, and 2) CIRRAL: the French National Institut of Cancer (10.13039/501100006364INCa), the French Association for Research in Cancer and the 10.13039/501100003323ANRS (PAIR CHC 2009). Pierre Nahon’s research is funded in part by the 10.13039/501100000780European Union (GENIAL, Grant agreement ID: 101096312), French 10.13039/501100001665Agence Nationale de la Recherche (France 2030 DELIVER ANR-21-RHUS-0001) and by France 2030 RHU LIVER-TRACK (ANR-23-RHUS-0014).

## Role of the sponsor

The funding sponsors had no role in the design and conduct of the study; the collection, management, analysis or interpretation of the data; or the preparation, review or approval of the manuscript.

## Conflicts of interest

Pr Nahon has received honoraria from and/or consults for AstraZeneca, Bristol-Myers Squibb, Eisai, and Roche. He received research grants from AstraZeneca, Bristol-Myers Squibb and Eisai. Pr Ganne-Carrié consults for and/or received personal fees from Abbvie, Bayer, Gilead, Ipsen, and Shionogi, outside the submitted work.

Please refer to the accompanying ICMJE disclosure forms for further details.
