# Supplementary material for: Comparative evaluation of regression and machine-learning models for hepatocellular carcinoma risk stratification across diverse aetiologies
Source: JHEP Rep. 2026 Feb 3;8(4):101740. doi: 10.1016/j.jhepr.2026.101740 (PMC12972975; doi:10.1016/j.jhepr.2026.101740)
Supplement: Multimedia component 1 [file mmc1.pdf]

Comparative evaluation of regression and machine-learning models for hepatocellular carcinoma risk stratification across diverse aetiologies

Pierre Nahon, Richard Layese, Pierre-André Natella, Lucia Parlati, Tounes Saidi, Nathalie Ganne-Carrié, Gisèle N'Kontchou, Cendrine Chaffaut, Jean-Charles Nault, Jessica Bamba-Funck, Angela Sutton, Clovis Lusivika Nzinga, Fabrice Carrat, Etienne Audureau, for the ANRS CO12 CirVir, ANRS CO22 Hepather, and CIRRAL groups

Table of contents

List of investigators…………………………………………………………..………2

Supplementary figures……………………………………………………………..…...5

Supplementary tables…………………………………………………….…….…...9

LIST OF INVESTIGATORS

ANRS CO12 CirVir group:
Pierre Nahon[1], Tarik Asselah[2], Dominique Guyader[3], Stanislas Pol[4], Hélène Fontaine[4], Georges-Philippe Pageaux[5], Victor De Lédinghen[6], Denis Ouzan[7], Fabien Zoulim[8], Dominique Roulot[9], Albert Tran[10], Jean-Pierre Bronowicki[11], Thomas Decaensi[12], Ghassan Riachi[13], Paul Calès[14], Jean-Marie Péron[15], Laurent Alric[16], Marc Bourlière[17], Philippe Mathurin[18], Sebastien Dharancy[18], Jean-Frédéric Blanc[19], Armand Abergel[20], Olivier Chazouillères[21], Ariane Mallat[22], Jean-Didier Grangé[23], Pierre Attali[24], Louis d'Alteroche[25], Claire Wartelle[26], Thông Dao[27], Dominique Thabut[28], Christophe Pilette[29], Christine Silvain[30], Christos Christidis[31], Eric Nguyen-Khac[32], Brigitte Bernard-Chabert[33], Sophie Hillaire[34], Vincent Di Martino[35].
[1]AP-HP, Hôpital Avicenne, Service d'Hépatologie, Bobigny, Université Paris 13, Bobigny et INSERM U1162, Université Paris 5, Paris; [2]AP-HP, Hôpital Beaujon, Service d'Hépatologie, and University Paris Diderot, Sorbonne Paris Cité, CRI, UMR 1149; [3]CHU Pontchaillou, Service d'Hépatologie, Rennes; [4]AP-HP, Hôpital Cochin, Département d'Hépatologie et INSERM UMS20 et U1223, Institut Pasteur, Université Paris Descartes, Paris; [5]Hôpital Saint Eloi, Service d'Hépatologie, Montpellier; [6]Hôpital Haut-Lévêque, Service d'Hépatologie, Bordeaux; [7]Institut Arnaud Tzanck, Service d'Hépatologie, St Laurent du Var; [8]Hôpital Hôtel Dieu, Service d'Hépatologie, Lyon; [9]AP-HP, Hôpital Avicenne, Service d'Hépatologie, Bobigny; [10]CHU de Nice, Service d'Hépatologie, et INSERM U1065, Université de Nice-Sophia-Antipolis, Nice; [11]Hôpital Brabois, Service d'Hépatologie, Vandoeuvre-les-Nancy; [12]Hôpital Michallon, Service d'Hépatologie, Grenoble; [13]Hôpital Charles-Nicolle, Service d'Hépatologie, Rouen; [14]CHU d'Angers, Service d'Hépatologie, Angers; [15]Hôpital Purpan, Service d'Hépatologie, Toulouse; [16]CHU Toulouse, Service de Médecine Interne-Pôle Digestif UMR 152, Toulouse; [17]Hôpital Saint Joseph, Service d'Hépatologie, Marseille; [18]Hôpital Claude Huriez, Service d'Hépatologie, Lille; [19]Hôpital St André, Service d'Hépatologie, Bordeaux; [20]Hôpital Hôtel Dieu, Service d'Hépatologie, Clermont-Ferrand; [21]AP-HP, Hôpital Saint-Antoine, Service d'Hépatologie, Paris; [22]AP-HP, Hôpital Henri Mondor, Service d'Hépatologie, Créteil; [23]AP-HP, Hôpital Tenon, Service d'Hépatologie, Paris; [24]AP-HP, Hôpital Paul Brousse, Service d'Hépatologie, Villejuif; [25]Hôpital Trousseau, Unité d'Hépatologie, CHRU de Tours; [26]Hôpital d'Aix-En-Provence, Service d'Hépatologie, Aix-En-Provence; [27]Hôpital de la Côte de Nacre, Service d'Hépatologie, Caen; [28]AP-HP, Groupe Hospitalier de La Pitié-Salpêtrière, Service d'Hépatologie, Paris; [29]CHU Le Mans, Service d'Hépatologie, Le Mans; [30]CHU de Poitiers, Service d'Hépatologie, Poitiers; [31]Institut Mutualiste Montsouris, Service d'Hépatologie, Paris; [32]Hôpital Amiens Nord, Service d'Hépatologie, Amiens; [33]Hôpital Robert Debré, Service d'Hépatologie, Reims; [34]Hôpital Foch, Service d'Hépatologie, Suresnes; [35]Hôpital Jean Minjoz, Service d'Hépatologie, Besançon. FRANCE.
Funding/Support: This study was sponsored by ANRS (France REcherche Nord & sud SIDA-HIV Hépatites: FRENSH).
This work is dedicated to the memory of Professor Jean-Claude Trinchet.

ANRS CO22 Hepather group:
Funding
INSERM-ANRS (France REcherche Nord&sud Sida-vih Hepatites), ANR (Agence Nationale de la Recherche), DGS (Direction Générale de la Santé) and MSD, Janssen, Gilead, Abbvie, BMS, Roche.
ANRS-AFEF Hepather Study group
Delphine Bonnet, Virginie Payssan-Sicart, Chloe Pomes (CHU Purpan, Toulouse, France), François Bailly, Marjolaine Beaudoin, Dominique Giboz, Kerstin Hartig-Lavie, Marianne Maynard (Hospices Civils de Lyon, Lyon, France), Eric Billaud, David Boutoille, Morane Cavellec, Marjorie Cheraud-Carpentier (Hôpital Hôtel-Dieu, Nantes, France), Isabelle Hubert, Jaouad Benhida, Adrien Lannes, Françoise Lunel, Frédéric Oberti (CHU Angers, Angers, France), Nathalie Boyer, Nathalie Giuily, Corinne Castelnau, Giovanna Scoazec (Hôpital Beaujon, Clichy, France), Aziza

Chibah, Sylvie Keser, Karim Bonardi, Anaïs Vallet-Pichard, Philippe Sogni (Hôpital Cochin, Paris, France), Juliette Foucher, Jean-Baptiste Hiriart, Amy Wilson, Sarah Shili, Faiza Chermak (Hôpital Haut-Lévêque, Pessac, Bordeaux, France), Christelle Ansaldi, Nisserine Ben Amara, Laëtitia Chouquet, Emilie De Luca, Valérie Oules (Hôpital Saint Joseph, Marseille, France), Rodolphe Anty, Eve Gelsi, Régine Truchi (CHU de Nice, Nice, France), Elena Luckina, Nadia Messaoudi, Joseph Moussali (Hôpital de la Pitié Salptétrière, Paris, France), Barbara De Dieuleveult, Damien Labarriere, Pascal Poter, Si Nafa Si Ahmed (CHR La Source, Orléans, France), Véronique Grando-Lemaire, Pierre Nahon, Valérie Bourcier, Séverine Brulé, Thomas Stalhberger (Hôpital Avicenne, Bobigny, France), Caroline Jezequel, Audrey Brener, Anne Laligant, Aline Rabot, Isabelle Renard (CHU Rennes, Rennes, France), Thomas F. Baumert, Michel Doffföel, Catherine Mutter, Pauline Simo-Noumbissie, Esma Razi (Hôpitaux Universitaires de Strasbourg, Strasbourg, France), Hélène Barraud, Mouni Bensenane, Abdelbasset Nani, Sarah Hassani-Nani, Marie-Albertine Bernard (CHU de Nancy, Nancy, France), Georges-Philippe Pageaux, Michael Bismuth, Ludovic Caillo, Stéphanie Faure, Marie Pierre Ripault (Hôpital Saint Eloi, Montpellier, France), Christophe Bureau, Jean Marie Peron, Marie Angèle Robic, Léa Tarallo (CHU Purpan, Toulouse, France), Marine Faure, Bruno Froissart, Marie-Noelle Hilleret, Jean-Pierre Zarski (CHU de Grenoble, Grenoble, France), Odile Goria, Victorien Grard, Hélène Montialoux (CHU Charles Nicolle, Rouen, France), Muriel François, Christian Ouedraogo, Christelle Pauleau, Anne Varault (Hôpital Henri Mondor, Créteil, France), Tony Andreani, Bénédicte Angoulevant, Azeline Chevance, Lawrence Serfaty (Hôpital Saint-Antoine, Paris, France), Teresa Antonini, Audrey Coilly, Jean-Charles Duclos Vallée, Mariagrazia Tateo (Hôpital Paul Brousse, Villejuif, France), Corinne Bonny, Chanteranne Brigitte, Géraldine Lamblin, Léon Muti (Hôpital Estaing, Clermont-Ferrand, France), Abdenour Babouri, Virginie Filipe (Centre Hospitalier Régional, Metz, France), Camille Barrault, Laurent Costes, Hervé Hagège, Soraya Merbah (Centre Hospitalier Intercommunal, Créteil, France), Paul Carrier, Maryline Debette-Gratien, Jérémie Jacques (CHU Limoges, Limoges, France), Guillaume Lassailly, Florent Artu, Valérie Canva, Sébastien Dharancy, Alexandre Louvet (CHRU Claude Huriez, Lille, France), Marianne Latournerie, Marc Bardou, Thomas Mouillot (Dijon University Hospital, Dijon, France), Yannick Bacq, Didier Barbereau, Charlotte Nicolas (CHU Trousseau, 37044 Tours, France), Caroline Chevalier, Isabelle Archambeaud, Sarah Habes (CHU de Nantes, Nantes, France), Nisserine Ben Amara, Danièle Botta-Fridlund, (CHU Timone, Marseille, France), Eric Saillard, Marie-Josée Lafrance, (CHU de Pointe-à-Pitre, Pointe-à-Pitre, Guadeloupe).

Scientific Committee:

- Voting members:

Marc Bourlière (Hôpital St Joseph, Marseille), Patrice Cacoub (Hôpital Pitié salpêtrière, Paris, France, Fabrice Carrat (Scientific Coordinator, Hôpital Saint-Antoine, Paris, France), Patrizia Carrieri (INSERM U912, Marseille, France), Elisabeth Delarocque-Astagneau (Inserm UMR1181, Paris), Victor De Ledinghen (Hôpital Haut-Lévêque, Pessac, Bordeaux, France), Céline Dorival (UPMC & INSERM U1136, Paris, France), Jean Dubuisson (Inserm U1019, Lille, France), Hélène Fontaine (Hôpital Cochin, Paris, France), Chantal Housset (Inserm UMR-S938 1 IFR65, Paris), Dominique Larrey (Hôpital Saint Eloi, Montpellier, France), Patrick Marcellin (Hôpital Beaujon, Clichy, France), Philippe Mathurin (CHRU Claude Huriez, Lille, France), Pierre Nahon (Hôpital Avicenne, Bobigny, France), Georges-Philippe Pageaux (Hôpital Saint Eloi, Montpellier, France), Jean-Michel Pawlotsky (Hôpital Henri Mondor, Créteil, France), Ventzislava Petrov-Sanchez (ANRS, Paris, France), Stanislas Pol (Principal Investigator, Hôpital Cochin, Paris, France), Sophie Vaux (Agence Nationale de Santé Publique, Saint Maurice, France), Linda Wittkop (ISPED-INSERM U897, Bordeaux, France), Yazdan Yazdanpanah (Hôpital Bichat Claude Bernard, Paris, France), Jean-Pierre Zarski (CHU de Grenoble, Grenoble, France), Fabien Zoulim (Hospices Civils de Lyon, Lyon, France), Jessica Zucman-Rossi (Inserm U674/1162, Paris).

- Nonvoting members:

Marianne L'hennaff (ARCAT-TRT-5-CHV, France), Michèle Sizorn (SOS hépatites, France); one representative of INSERM-ANRS Pharmacovigilance team, Paris, France (Imane Amri, Alpha

Diallo), Mélanie Simony, Carole Cagnot (INSERM-ANRS, Paris, France), one member of Inserm Transfert, Paris, France (Alice Bousselet, Mireille Caralp, Jean-Marc Lacombe), and one representative of each pharmaceutical company (MSD, Janssen, Gilead, Abbvie, BMS, Roche).
Sponsor: Imane Amri, Alpha Diallo, Carole Cagnot, Mélanie Simony (INSERM-ANRS, Paris, France), Ventzi Petrov-Sanchez (coordinator).
Methodology and Coordinating Centre: Loubna Ayour, Jaouad Benhida, Fabrice Carrat (coordinator), Frederic Chau, Céline Dorival, Audrey Gilibert, Isabelle Goderel, Victorien Grard, Warda Hadi, Georges Haour, Godwin Mawuvi, Léa Mba Mintsa, Grégory Pannetier, François Pinot, Muriel Sudres, François Téloulé (Sorbonne Université & INSERM U1136, Paris, France).

CIRRAL group:
Nathalie Ganne-Carrié[1,], Cendrine Chaffaut[2], Isabelle Archambeaud[3], Louis d'Alteroche[4], Frédéric Oberti[5], Dominique Roulot[6], Christophe Moreno[7], Alexandre Louvet[8], Thông Dao[9], Romain Moirand[10], Odile Goria[11], Eric Nguyen-Khac[12], Nicolas Carbonell[13], Jean-Charles Duclos-Vallée[14], Stanislas Pol[15], Victor de Ledinghen[16], Violaine Ozenne[17], Jean Henrion[18], Jean-Marie Péron[19], Albert Tran[20], Gabriel Perlemuter[21], Xavier Amiot[22], Jean-Pierre Zarski[23], Sylvie Chevret[2].
[1]AP-HP, Hôpital Avicenne, Service d'Hépatologie, Bobigny, Université Sorbonne Paris Nord, Bobigny et INSERM U1138, Université de Paris; [2]SBIM, APHP, Hôpital Saint-Louis, Paris, Inserm, UMR-1153, ECSTRA Team, Paris, France; [3]Liver, CHU, Nantes, France; [4]Liver Unit, University Hospital, Tours, France; [5]Liver Unit, University Hospital, Angers, France; [6]AP-HP, Hôpital Avicenne, Service de Médecine Interne, Bobigny, Université Sorbonne Paris Nord, Bobigny; [7]Liver unit, CUB Hôpital Erasme, Université Libre de Bruxelles, Belgium, [8]Liver Unit, University Hospital, Lille, France, [9]Liver Unit, University Hospital, Caen, France ; [10]Liver Unit, University Hospital, Rennes, France ; [11]Liver Unit, University Hospital, Rouen, France ; [12]Liver Unit, University Hospital, Amiens, France ; [13] Liver Unit, APHP, CHU Saint-Antoine, Paris, France ; [14]Liver Unit, APHP, CHU Paul Brousse, Villejuif, France; [15]Université Paris Descartes; APHP, Liver Unit, Hôpital Cochin; INSERM U1223, Institut Pasteur, Paris, France; [16]Hepatology Unit, University Hospital, CHU Bordeaux, France; [17]Liver Unit, APHP, CHU Lariboisière, Paris, France; [18]Liver Unit, University Hospital, Haine Saint-Paul, Belgium, [19]Liver Unit, Universitary Hospital Purpan, University Paul Sabatier III, Toulouse ; [20]Institut National de la Santé et de la Recherche Médicale (INSERM), U1065, Team 8, "Hepatic Complications in Obesity", Nice, F-06204, Cedex 3, France, University Hospital of Nice, Digestive Centre, Nice, F-06202, Cedex 3, France ; [231]Liver Unit, University Hospital, Béclère, APHP, Clamart, France; [22]Liver Unit, APHP, CHU Tenon, Paris, France; [23]Clinique d'hépato-gastroentérologie pôle Digidune CHU de Grenoble, France
Funding/Support: The promoter of the study was the Assistance Publique des Hôpitaux de Paris (APHP), and the cohort was funded by the Institut National du Cancer (INCa).
Role of the Sponsor: The funding sponsor had no role in the design and conduct of the study; collection, management, analysis, interpretation of the data, and preparation, review, or approval of the manuscript.

Supplementary figures

Fig. S1. Constitution of the training and validation sets. Centers from the CirVir (A_1…A_l), Cirral (B_1…B_m), and Hepather (C_1…C_n) cohorts were pooled to create the total study population. This population was then split into a training set (approximately two-thirds of patients) and a validation set (the remaining one-third). To preserve center-level independence, all patients from a given center were randomly assigned together to either the training or the validation set.

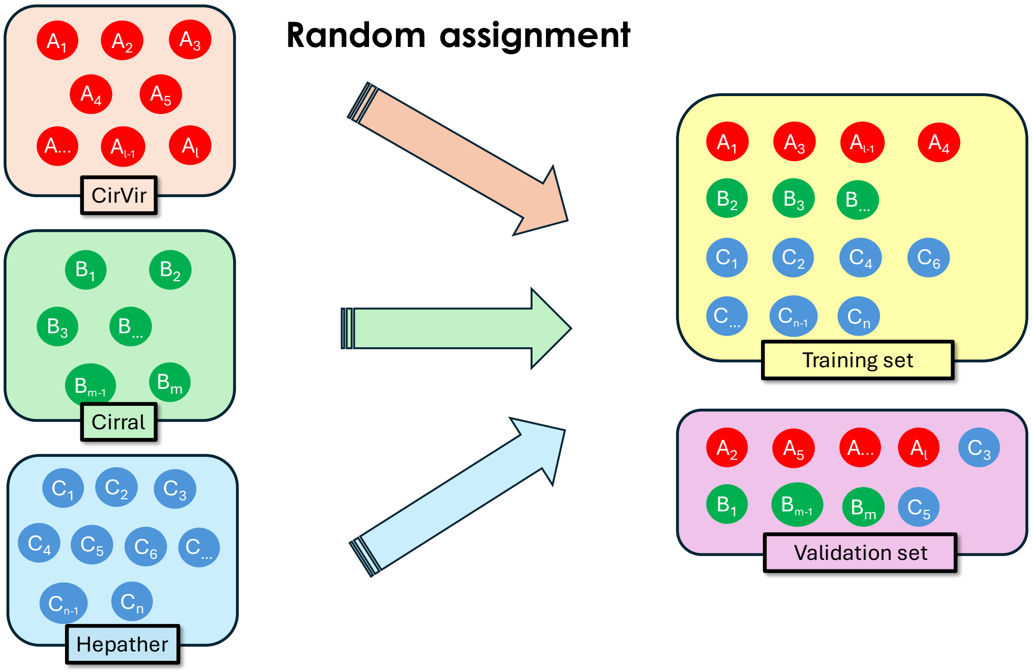

Fig. S2. Out of bag error rate against number of trees. A minimal number of 1000 trees is required to reach stability of the Out-Of-Bag error rate.

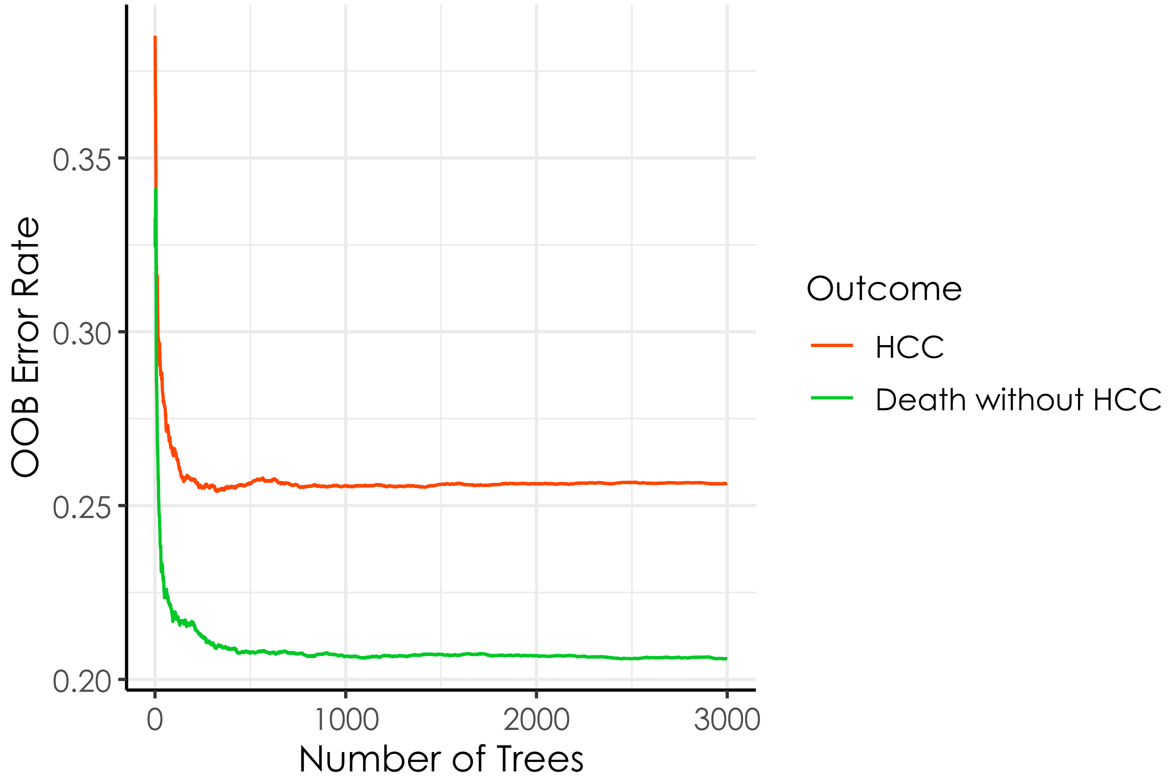

Fig. S3. Flowchart

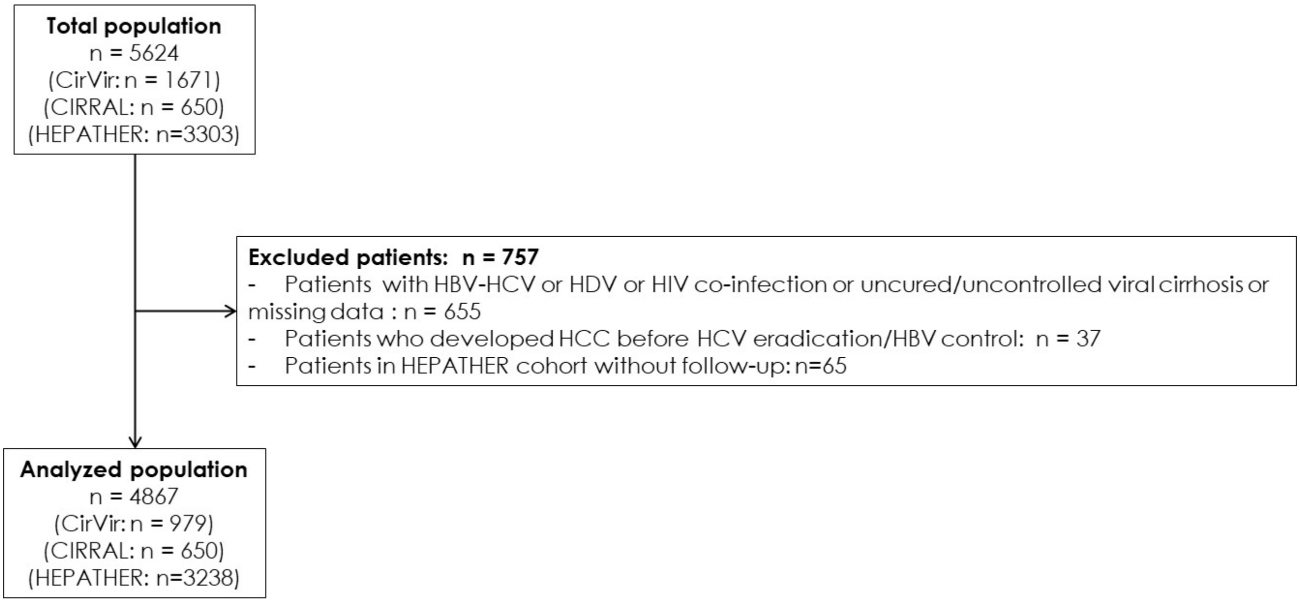

Fig. S4. HCC incidence in the development (n=3251) and validation (n=1616) cohorts. Levels of significance: P<0.05 (*Gray's test)*

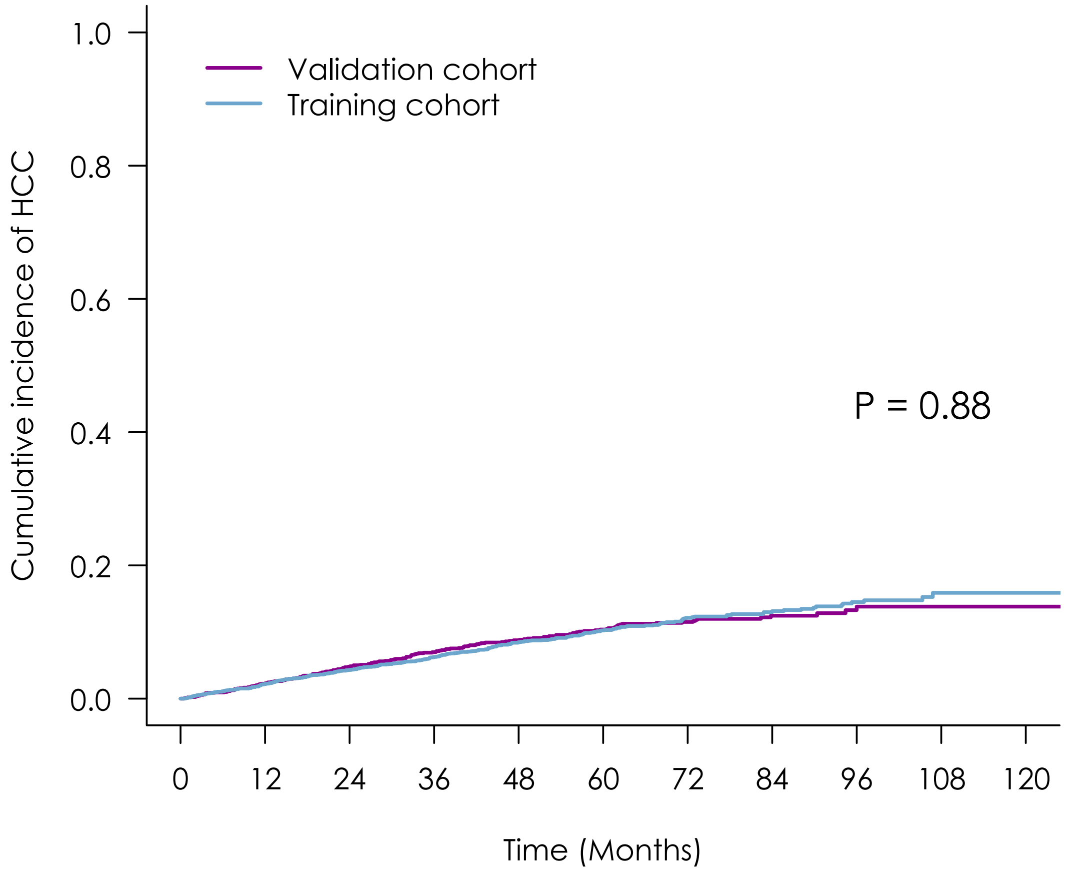

Supplementary tables

Table S1. Baseline characteristics of each cohort

**Characteristic**	**N**	**Overall** N = 4867[1]	**CIRRAL** N=650[1]	**CIRVIR** N=979[1]	**HEPATHER** N=3238[1]	**p-value**[2]
Age	4867	58.1 [52.0;66.0]	58.0 [51.0;64.0]	57.0 [50.0;65.0]	58.8 [52.5;66.9]	**<0.001**
Male sex		3,280 (67.4%)	438 (67.4)	669 (68.3)	2,173 (67.1)	
Platelet count, $10^3/mm^3$	4223	160 [113;208]	145 [104;191]	159 [110;202]	165 [117;213]	**<0.001**
AST	4259	29.0 [23.0;39.0]	33.0 [26.0;49.0]	31.0 [25.0;41.0]	28.0 [22.0;36.5]	**<0.001**
ALT	4397	26.0 [19.0;38.0]	26.0 [20.0;39.0]	29.0 [21.0;43.0]	25.0 [19.0;36.0]	**<0.001**
GGT	4082	45.0 [26.0;93.0]	108 [52.0;217]	42.0 [25.0;73.0]	39.0 [24.0;74.0]	**<0.001**
Prothrombin time, %	2955	87.0 [76.0;97.0]	78.0 [67.0;90.0]	89.0 [81.0;99.0]	88.0 [78.0;97.0]	**<0.001**
Serum albumin, g/L	3352	42.0 [38.9;45.0]	40.5 [37.0;43.2]	43.0 [40.0;46.0]	42.0 [39.0;45.0]	**<0.001**
Total bilirubin, µmol/L	3578	11.0 [8.0;16.1]	14.0 [10.0;20.0]	10.0 [7.0;14.0]	11.0 [8.0;16.0]	**<0.001**
Alpha-fetoprotein, ng/mL	3842	5.2 [3.00;10.4]	3.80 [2.50;5.5]	3.00 [2.00;5.0]	7.0 [3.80;13.7]	**<0.001**
INR	2685	1.10 [1.02;1.20]	1.20 [1.10;1.30]	1.10 [1.10;1.14]	1.10 [1.02;1.20]	**<0.001**
Creatinin	3139	71.0 [61.3;83.6]	70.0 [60.0;82.7]	74.0 [63.0;86.0]	70.7 [61.3;82.7]	**<0.001**
Glycemia	2851	1.03 [0.90;1.60]	5.6 [5.00;6.7]	0.99 [0.88;1.10]	0.99 [0.89;1.18]	**<0.001**
Alcalines phospatases	4019	88.0 [68.0;121]	96.0 [73.0;133]	83.5 [65.0;116]	88.8 [69.0;118]	**<0.001**
Ferritin	4867	233 [120;427]	112 [68.9;215]	234 [122;467]	260 [142;444]	**<0.001**
Cirrhosis aetiology	4867					
Cured HCV		3,543 (72.8%)	0 (0.0)	742 (75.8)	2,801 (86.5)	
Controlled HBV		674 (13.8%)	0 (0.0)	237 (24.2)	437 (13.5)	
Alcohol and/or Met-ALD		650 (13.4%)	650 (100.0)	0 (0.0)	0 (0.0)	

[1]Median [Q1;Q3]; n (%)

[2]Kruskal-Wallis rank sum test; Fisher's exact test. *Levels of significance: P<0.05*

Table S2. Discrimination performance indices by cohort (validation sets)

Time-dependent AUC	CirVir	CIRRAL	HEPATHER
At 1 year			
aMAP score	0.7641 (0.6528 ; 0.8755)	0.8505 (0.6493 ; 1.00)	0.6979 (0.5960 ; 0.7998)
FASTRAK score	0.7127 (0.5229 ; 0.8995)	0.8262 (0.6336 ; 1.00)	0.7136 (0.6146 ; 0.8125)
Decision tree	0.6066 (0.3956 ; 0.8176)	0.5815 (0.3435 ; 0.8195)	0.7110 (0.6046 ; 0.8174)
Survival random forest	0.7393 (0.5815 ; 0.8971)	0.6809 (0.4866 ; 0.8752)	0.7594 (0.6647 ; 0.8541)
Deep neural network	0.7694 (0.6605 ; 0.8783)	0.7544 (0.5826 ; 0.9261)	0.7534 (0.6481 ; 0.8583)
At 3 years			
aMAP score	0.6782 (0.5163 ; 0.8401)	0.9198 (0.8618 ; 0.9777)	0.6475 (0.5805 ; 0.7145)
FASTRAK score	0.6735 (0.5475 ; 0.7995)	0.8469 (0.7741 ; 0.9197)	0.7007 (0.6366 ; 0.7648)
Decision tree	0.6073 (0.4740 ; 0.7406)	0.6748 (0.5373 ; 0.8123)	0.6785 (0.6143 ; 0.7426)
Survival random forest	0.6748 (0.5667 ; 0.7829)	0.7783 (0.6951 ; 0.8616)	0.7281 (0.6670 ; 0.7891)
Deep neural network	0.7427 (0.6492 ; 0.8362)	0.8623 (0.8010 ; 0.9235)	0.6996 (0.6323 ; 0.7670)
At 5 years			
aMAP score	0.7326 (0.5994 ; 0.8659)	0.8523 (0.7293 ; 0.9753)	0.6234 (0.5667 ; 0.6800)
FASTRAK score	0.7199 (0.6190 ; 0.8209)	0.8201 (0.7359 ; 0.9043)	0.6558 (0.5962 ; 0.7155)
Decision tree	0.6246 (0.5019 ; 0.7474)	0.6558 (0.5341 ; 0.7775)	0.6483 (0.5923 ; 0.7042)
Survival random forest	0.7084 (0.6145 ; 0.8022)	0.7363 (0.6453 ; 0.8273)	0.6733 (0.6123 ; 0.7343)
Deep neural network	0.7973 (0.7109 ; 0.8837)	0.7543 (0.6402 ; 0.8684)	0.6669 (0.6049 ; 0.7289)

Brier score	CirVir	CIRRAL	HEPATHER
At 1 year			
aMAP score	0.019 (0.004 ; 0.034)	0.016 (0.001 ; 0.031)	0.023 (0.015 ; 0.032)
FASTRAK score	0.019 (0.004 ; 0.034)	0.016 (0.001 ; 0.061)	0.023 (0.015 ; 0.032)
Decision tree	0.019 (0.005 ; 0.034)	0.018 (0.002 ; 0.033)	0.023 (0.014 ; 0.032)
Survival random forest	0.019 (0.004 ; 0.035)	0.017 (0.002 ; 0.033)	0.023 (0.014 ; 0.032)
Deep neural network	0.019 (0.005 ; 0.034)	0.018 (0.004 ; 0.033)	0.023 (0.015 ; 0.031)
At 3 years			
aMAP score	0.052 (0.028 ; 0.076)	0.058 (0.032 ; 0.084)	0.065 (0.051 ; 0.078)
FASTRAK score	0.052 (0.028 ; 0.076)	0.060 (0.034 ; 0.085)	0.063 (0.050 ; 0.076)
Decision tree	0.052 (0.028 ; 0.077)	0.065 (0.039 ; 0.092)	0.063 (0.050 ; 0.077)
Survival random forest	0.051 (0.027 ; 0.074)	0.066 (0.038 ; 0.093)	0.063 (0.049 ; 0.076)
Deep neural network	0.052 (0.030 ; 0.074)	0.063 (0.039 ; 0.086)	0.063 (0.051 ; 0.075)
At 5 years			
aMAP score	0.072 (0.044 ; 0.100)	0.084 (0.052 ; 0.115)	0.092 (0.077 ; 0.107)
FASTRAK score	0.073 (0.044 ; 0.102)	0.087 (0.056 ; 0.117)	0.089 (0.074 ; 0.103)
Decision tree	0.076 (0.048 ; 0.104)	0.096 (0.066 ; 0.125)	0.089 (0.074 ; 0.103)
Survival random forest	0.073 (0.046 ; 0.101)	0.094 (0.063 ; 0.125)	0.088 (0.073 ; 0.102)
Deep neural network	0.075 (0.052 ; 0.098)	0.097 (0.071 ; 0.124)	0.092 (0.079 ; 0.104)
