# Supplementary material for: Comparative evaluation of regression and machine-learning models for hepatocellular carcinoma risk stratification across diverse aetiologies
Source: JHEP Rep. 2026 Feb 3;8(4):101740. doi: 10.1016/j.jhepr.2026.101740 (PMC12972975; doi:10.1016/j.jhepr.2026.101740)
Supplement: Multimedia component 4 [file mmc4.pdf]

Research article

JHEP|Reports

Comparative evaluation of regression and machine-learning models for hepatocellular carcinoma risk stratification across diverse aetiologies

Authors

Pierre Nahon, Richard Layese, Pierre-André Natella, …, Clovis Lusivika Nzinga, Fabrice Carrat, Etienne Audureau

Correspondence

pierre.nahon@aphp.fr (P. Nahon).

Graphical abstract

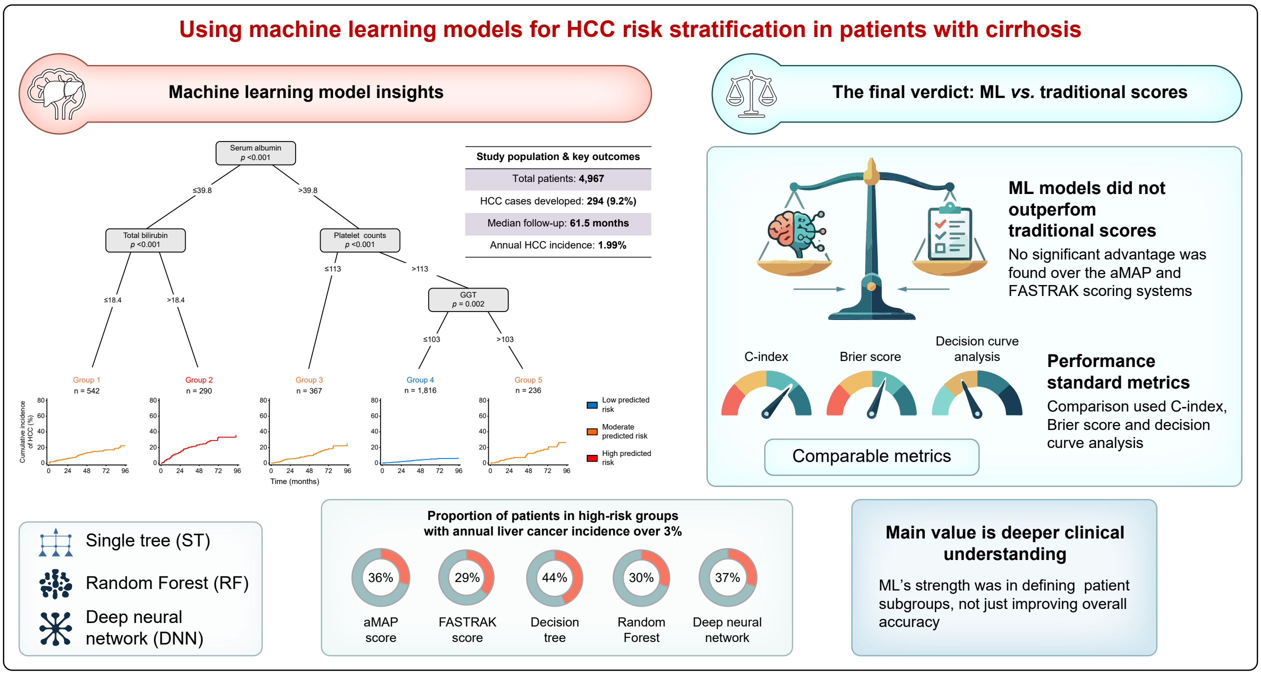

Highlights:

- ML models were developed to stratify HCC risk in patients with cirrhosis.
- A simple decision tree identified four predictors and defined five clinically meaningful HCC risk groups.
- High-risk groups (annual HCC risk >3%) accounted for 44% of patients.
- Random forest and deep neuronal network approaches confirmed complex predictor interactions.
- ML models did not outperform clinical scores but improved clinical interpretability.

Impact and implications:

Accurate stratification of hepatocellular carcinoma risk in cirrhosis is essential to optimize surveillance strategies, and this study provides a scientific rationale for exploring machine learning approaches to capture complex, non-linear interactions among clinical variables beyond traditional regression models. Although machine learning did not improve predictive performance over established scores, it revealed clinically meaningful risk subgroups defined by liver function, platelet count, and GGT, underscoring its value as an interpretative and hypothesis-generating tool. These results are particularly relevant for hepatologists and clinical researchers seeking to refine risk-adapted surveillance and to inform the design of future models or trials.

https://doi.org/10.1016/j.jhepr.2026.101740
© 2026 The Author(s). Published by Elsevier B.V. on behalf of European Association for the Study of the Liver (EASL). This is an open access article under the CC BY license (http://creativecommons.org/licenses/by/4.0/).

JHEP|Reports

Comparative evaluation of regression and machine-learning models for hepatocellular carcinoma risk stratification across diverse aetiologies

Pierre Nahon[1,*], Richard Layese[2], Pierre-André Natella[2], Lucia Parlati[3], Tounes Saidi[4], Nathalie Ganne-Carrié[1], Gisèle N'Kontchou[1], Cendrine Chaffaut[5], Jean-Charles Nault[1], Jessica Bamba-Funck[6], Angela Sutton[6], Clovis Lusivika Nzinga[7], Fabrice Carrat[7], Etienne Audureau[2], for the ANRS CO12 CirVir, ANRS CO22 Hepather, and CIRRAL groups

JHEP Reports **2026**. vol. 8 | 1–11

Check for updates

Background & Aims: We aimed to develop machine learning (ML) models for hepatocellular carcinoma (HCC) risk stratification in patients with cirrhosis and to test their ability to identify those with an annual HCC incidence >3%, for whom more intensive surveillance may be justified.

Methods: Data from three prospective cohorts (ANRS CO12 CirVir, CO22 Hepather, APHP CIRRAL) were analyzed. All patients underwent semiannual ultrasound surveillance and were randomly split into training and validation sets. HCC incidence was evaluated using a competing risk framework. A single tree (ST) model was developed using conditional decision trees, while random forest (RF) models were built by aggregating 1,000 trees. A deep neural network (DNN)–based survival model was also applied. ML model performance was compared with established regression-based scores: aMAP (age–male–ALBI–platelets) and FASTRAK (FAST-MRI for HCC suRveillance in pAtients with high risK of liver cancer).

Results: Among 4,867 patients with non-viral cirrhosis or resolved/controlled viral cirrhosis, 294 (9.2%) developed HCC over a median follow-up of 61.5 months (annual incidence: 1.99%). The ST model identified four key predictors, generating five distinct risk groups. These included patients with mildly impaired liver function or those with elevated GGT and low platelet counts. The RF and DNN approaches confirmed ST findings and delineated complex interactions among predictors. Performance metrics (C-index, Brier score, decision curve analysis) showed no significant advantage of ML models over aMAP and FASTRAK. Calibration was consistent across models. ML models identified higher proportions of patients with an annual HCC incidence >3% (ST 44%; DNN 37%; RF 30%) compared with aMAP (36%) and FASTRAK (29%).

Conclusions: ML-based algorithms did not outperform traditional risk scores but provided novel insights into variable interactions and helped identify clinically relevant patient subgroups with differing HCC risk profiles.

© 2026 The Author(s). Published by Elsevier B.V. on behalf of European Association for the Study of the Liver (EASL). This is an open access article under the CC BY license (http://creativecommons.org/licenses/by/4.0/).

Introduction

Early detection of hepatocellular carcinoma (HCC) through regular surveillance allows for the application of curative treatments, significantly improving outcomes for patients with cirrhosis or advanced chronic liver disease (ACLD).[1] Although semiannual liver ultrasound remains the recommended primary imaging method due to its accessibility and safety, advanced diagnostics such as circulating biomarkers and specialized liver MRI protocols are under investigation for their enhanced diagnostic accuracy.[2,3] However, these more costly and less widely available tools may only be cost-effective for patients with the highest risk of liver cancer, particularly those with an annual incidence exceeding 3%.[4]

The cost-effectiveness of surveillance strategies is largely influenced by HCC incidence, the aetiologies of which have shifted considerably over the past decade.[5] Alcohol use and metabolic-associated steatotic liver disease (MASLD) have become the leading risk factors for cirrhosis and HCC,[6–8] while the prevalence of untreated HCV and HBV infections has significantly declined.[9] These trends have led to a reduced overall risk of HCC among patients with cirrhosis compared to historical cohorts, with current annual incidence rates ranging from 1% to 2% in patients with compensated non-viral ACLD or controlled HBV/cured HCV infections.[8,10–12]

Several risk models and scoring systems have been designed to assess the likelihood of HCC development in patients with cirrhosis, typically integrating clinical, laboratory, and demographic factors to estimate individual risk.[3,13] These scoring systems may in the near future assist clinicians in identifying individuals at higher risk of developing HCC, thus enabling targeted surveillance strategies and interventions for early detection that are currently being tested in randomized

* Corresponding author. Address: Service d'Hépatologie, Hôpital Avicenne, 93000 Bobigny, France; Tel.: 33-1-48-02-62-80, fax: 33-1-48-02-62-02.
E-mail address: pierre.nahon@aphp.fr (P. Nahon).
https://doi.org/10.1016/j.jhepr.2026.101740

trials.[3,14] More recently, machine learning (ML) approaches have shown promise in risk stratification by utilizing various clinical, laboratory, imaging, and genetic data to predict an individual's risk of developing a given disease.[15] ML algorithms, including those based on decision trees, have the theoretical capability of detecting intricate interactions among predictive factors and thus help identifying more specific high-risk subgroups. By delivering more accurate personalized risk assessments, ML approaches have the potential to enhance HCC risk stratification, promote early detection, and improve patient outcomes.

This study aimed to develop ML models for HCC risk stratification in large prospective cohorts of patients with cirrhosis, taking into account recent changes in the ACLD epidemiological landscape, and to evaluate their relative performance against scoring systems based on conventional biostatistical approaches.

Patients and methods

Patients

The present work used data from one randomized clinical trial dedicated to HCC surveillance and three French prospective cohorts of adults with biopsy-proven compensated cirrhosis without detectable suspected focal liver lesions: the HCC 2000 trial,[16] the ANRS CO12 CirVir cohort,[17] the CIRRAL cohort,[12] and the ANRS CO22 Hepather cohort.[18] Each study was conducted in accordance with the ethical guidelines of the 1975 Declaration of Helsinki and French laws for biomedical research and was approved by Ethics Committees. All patients gave written informed consent to participate.

All patients enrolled in these cohorts underwent periodic liver ultrasound surveillance in accordance with international and French guidelines, with or without measurement of serum alpha-fetoprotein (AFP) levels. In cases where focal liver lesions were detected, a recall diagnostic work-up using contrast-enhanced imaging (CT or MRI) and/or image-guided biopsy was performed in accordance with the 2005 AASLD guidelines, updated in 2011.[19,20] A diagnosis of HCC was thus established by either histological examination or based on probabilistic non-invasive criteria (mainly dynamic imaging revealing early arterial hyperenhancement and washout on portal venous or delayed phases) according to the different time periods (before and after 2011). When HCC diagnosis was established, treatment was determined using a multidisciplinary approach according to AASLD[19,20] and the EASL–EORTC[21] guidelines.

In addition to HCC occurrence, which was the primary endpoint of all four cohorts, all events that occurred during follow-up (*i.e.* death, liver decompensation,[22] bacterial infection,[23] extrahepatic malignancies[24] and cardiovascular diseases[25]) were recorded using information obtained from the medical records of patients held by each centre. Moreover, likely cause(s) of death were established. Patients who underwent liver transplantation were censored for analysis at the date of transplantation. All treatments, including antiviral therapies, were recorded at inclusion, and patients were notified of any modifications during follow-up. A single database encompassing clinical data from the four cohorts was built on November 18, 2019.[26] Among all included patients, only those with non-viral causes of cirrhosis (alcohol- and/or MASLD-related) or those who achieved HBV control/HCV eradication during follow-up were considered for the present analyses.

ANRS CO12 CirVir cohort

The ANRS CO12 CirVir cohort, sponsored and funded by the ANRS (France REcherche Nord & Sud Sida-HIV Hépatites), is a multicentre observational cohort that aims to characterize the incidence of complications occurring in biopsy-proven compensated cirrhosis and to identify the associated risk factors using competing risks analysis.[17] The full CirVir protocol is available via the ANRS Web site (http://anrs.fr). Specific additional inclusion criteria were i) cause of cirrhosis related to either chronic infection with HCV and/or HBV regardless of the levels of replication and alcohol consumption, ii) patients belonging to Child-Pugh A at enrolment, iii) absence of previous hepatic complications (particularly ascites, gastrointestinal haemorrhage, or HCC), and iv) absence of severe uncontrolled extrahepatic disease resulting in an estimated life expectancy of less than 1 year.

Among 1,822 patients recruited in 35 French clinical centres between March 2006 and July 2012, 151 were subsequently excluded from analysis after reviewing individual data due to either non-compliance with inclusion criteria (n = 142) or consent withdrawal (n = 9), leading to a total of 1,671 patients selected for further analysis, including the present study.

CIRRAL cohort

CIRRAL is a multicentre cohort study implemented in 22 French and 2 Belgian tertiary liver centres to capture the whole spectrum of complications occurring in compensated alcohol-related cirrhosis (complicated or not by metabolic syndrome) using competing risk analyses.[12] The promoter was the APHP. The cohort was funded by the French National Institute of Cancer (INCa), the French Association for Research in Cancer and the ANRS (PAIR CHC 2009) and was registered on ClinicalTrials.gov (NCT00190385). Specific additional inclusion criteria were i) cause of cirrhosis related to chronic alcohol abuse according to the World Health Organization criteria (more than 21 glasses per week for females and more than 28 glasses per week for males) for at least 10 years, ii) absence of chronic infection with HCV or HBV, and iii) patients belonging to Child-Pugh A at enrolment. The follow-up of patients was strictly superposed to the ANRSCO12Cirvir cohort design.

Among 706 patients included between October 2010 and April 2016, 54 were subsequently excluded after reviewing individual data because of violations of the inclusion criteria (n = 48) or consent withdrawal (n = 6); ultimately, a total of 652 patients were selected for further analysis, including the present study.

ANRS CO22 Hepather cohort

The ANRS CO22 Hepather cohort is a French national, multicentre, prospective, observational cohort study of patients with HBV or HCV infection that started in August 2012, among whom 3,045 had active HCV-related cirrhosis at inclusion.[18] Among the latter, a subset of 1,374 patients consecutively enrolled between 08/2012 and 01/2014 who responded to similar inclusion criteria as those included in the CirVir and CIRRAL cohorts were selected. Follow-up, antiviral treatments,

and the definition of the endpoint were identical to those in the CirVir cohort.

Endpoint

HCC cumulative incidence was the primary endpoint for predictive modelling. The baseline was defined as the date of inclusion in the corresponding cohort for patients with non-viral causes of cirrhosis and the date of sustained virological response achievement/viral control for patients with HCV/HBV-related cirrhosis. All analyses were conducted under a competing risk framework, considering death as a competing event for HCC occurrence.

Statistical analyses

Descriptive statistics

Descriptive results are presented as medians (IQR) for continuous variables and as numbers (percentages) for categorical data. Comparison of characteristics between groups were performed using the Mann-Whitney rank-sum test for continuous variables and the chi-squared test or Fisher's exact test for categorical variables.

Predictive modelling

To fulfil the primary objective of this analysis, different modelling approaches for HCC risk stratification were developed and validated using the three aforementioned cohorts. To ensure appropriate separation between development and validation data, each of the three cohorts was split into a training set (two thirds of patients) and a validation set (one third) using center-based random sampling. Entire centers were randomly assigned to either the training or the validation dataset so that no center contributed patients to both sets. Following this center-level split, missing data in the training and validation sets were imputed independently using the missForest algorithm to avoid any risk of information leakage. Fig. S1 describes the methodology used for the constitution of training and validation sets.

Several modeling approaches to HCC prediction were implemented and compared for their predictive performance and clinical significance. First, decision tree-based ML methods were applied. The models included previously identified and validated risk factors for HCC, commonly used in traditional risk scoring systems based on multivariate Cox proportional hazards modeling. These variables included age, sex, markers of liver condition (serum aspartate aminotransferase, alanine aminotransferase, gamma-glutamyltransferase [GGT], AFP), and surrogate indicators of portal hypertension (platelet count) or liver failure (prothrombin time, total and direct bilirubin levels). In addition to these established HCC risk factors, the ML models also incorporated routine biological parameters that were consistently available in all patient medical records: international normalized ratio, serum creatinine, blood glucose (glycemia), alkaline phosphatase, and serum ferritin.

For illustrative purposes, a single decision tree (ST) was initially built by recursive partitioning analysis using the conditional inference tree methodology,[27] because of its visual appeal to display the main relationships at play. Starting with all observations, the algorithm automatically identifies the optimal splits in data to partition the population into subgroups with differentiated HCC risks, repeating the process recursively until a stopping criterion is met.

Because of the ST approach's tendency for overfitting the training data, we then derived prognostic algorithms using a random (survival) forest (RF) approach for censored data, taking into account a competing risks framework. RFs combine the results obtained from a large ensemble of trees, thus avoiding the problem of selecting a single tree of appropriate size and often producing more stable predictive models.[28] In the present analysis, we used 1,000 trees, selected based on an examination of the out-of-bag error rate across a range of 100–3,000 trees. The out-of-bag error rate stabilized around 1,000 trees, with no meaningful improvement in model performance beyond this point (see Fig. S2). Unlike Fine-Gray modeling or single decision trees, RFs do not produce regression coefficients or decision paths to enable direct interpretation of the complex underlying prognostic model, so they are sometimes viewed as "black-boxes". Variable importance measures were thus computed to help quantify the importance of each predictor within the RF, by examining the increase in prediction error when a perturbation is added to the variable. To optimize the RF model, we used the mlr3 R package to select the most important features to keep in the model[29] and to perform hyperparameter tuning using a grid search with internal five-fold cross-validation on the development set. Optimal values for the mtry (range 2–50) and node size (range 2–50) parameters – corresponding to the number of variables sampled at each split and the degree of pruning in each decision tree, respectively – were identified.

Finally, we implemented a deep neural network (DNN)-based survival model to estimate survival probabilities without relying on the proportional hazards assumption, using the DeepHit framework.[30] DeepHit can accommodate non-linear and potentially non-proportional relationships between covariates and event times and explicitly models competing risks by predicting the joint distribution of survival times and event types, thereby offering greater flexibility. Hyperparameters were optimized using Optuna, a Bayesian optimization framework.

The performances of the machine learning models were compared to those of two previously published HCC risk scores, both derived using multivariate Cox proportional hazards modeling or Fine-Gray modeling and developed in contemporary cohorts of patients with ACLD without active viral replication.

The first was the age–male–ALBI–platelets (aMAP) score,[31] which includes age, sex, ALBI score, and platelet count. The second was the FASTRAK score, previously developed by our group in a subset of the present population under study,[4] and currently used in an ongoing randomized controlled trial[14] investigating the addition of FAST-MRI in high-risk patients with cirrhosis, defined by an annual HCC incidence >3% (FASTRAK trial, NCT05095714; FAST-MRI for HCC suRveillance in pAtients with high risK of liver cancer). This score incorporates age, sex, platelet count, GGT, total bilirubin, and AFP.

For all modeling approaches, risk classes were generated based on quintiles of the predicted 3-year HCC risk score. The discriminative performance of the prognostic models was assessed in the validation set using time-dependent AUC[32] and Brier score, which measure the probability of concordance

Table 1. Baseline characteristics of the studied population.

	Available data, n	Development cohort n = 3,251	Validation cohort n = 1,616	ASMD*	*p* value
Age	4,867	59.1 ± 10.7<br>58.7 [52.0–66.0]	58.8 ± 10.9<br>58.0 [51.7–66.0]	0.028	0.35
Male sex	4,867	2196 (67.5)	1084 (67.1)	0.010	0.75
Platelet count, $10^3/mm^3$	4,223	161 [115–210]	159 [109–205]	0.032	0.26
AST	4,259	29.0 [23.0–39.0]	29.0 [23.0–39.0]	0.014	0.93
ALT	4,397	26.0 [19.0–38.0]	26.0 [19.0–38.0]	0.009	0.43
GGT	4,082	45.0 [26.0–91.0]	45.0 [27.8–95.0]	0.055	0.14
Prothrombin time, %	2,955	87.0 [76.0–97.0]	86.0 [76.0–95.0]	0.120	**0.007**
Serum albumin, G/L	3,352	42.0 [38.8–45.0]	42.0 [39.0–45.0]	0.043	0.79
Total bilirubin, μmol/L	3,578	11.0 [8.0–16.0]	11.0 [8.0–16.2]	0.010	0.70
Alpha-fetoprotein, ng/ml	3,842	5.1 [3.00–10.5]	5.2 [3.00–10.2]	0.009	0.84
INR	2,685	1.10 [1.01–1.20]	1.10 [1.05–1.19]	0.025	0.15
Creatinine	3,139	71.0 [61.3–83.1]	71.6 [61.9–84.6]	0.037	0.25
Glycemia	2,851	1.03 [0.90–1.60]	1.04 [0.90–1.64]	0.008	0.46
Alkaline phosphatase	4,019	89.0 [69.0–124]	86.5 [67.0–114]	0.118	**0.002**
Ferritin	4,867	239 [107–491]	235 [100–502]	0.036	0.75
Cirrhosis aetiology	4,867				0.065
Cured HCV		2,377 (73.1%)	1,166 (72.2%)	0.022	
Controlled HBV		464 (14.3%)	210 (13.0%)	0.007	
Alcohol and/or metabolic		410 (12.6%)	240 (14.9%)	0.041	
Cohorts	4,867				0.094
CIRRAL		410 (12.6%)	240 (14.9%)	0.065	
CirVir		657 (20.2%)	322 (19.9%)	0.037	
HEPATHER		2,184 (67.2%)	1,054 (65.2%)	0.065	

ALT, alanine aminotransferase; ASMD, absolute standardized mean difference; AST, aspartate aminotransferase; GGT, gamma-glutamyltransferase; INR, international normalized ratio.
Results are given as n (%) for categorical variables and mean (±SD) or median (IQR) for continuous variables.
*A value of ASMD >0.10 denotes an imbalance of the studied parameter between the two groups.

between predicted and observed survival,[33] along with their confidence intervals. We also estimated the standardized 'net benefit' derived from decision curve analysis[34,35]), a method that is increasingly used for evaluating alternative diagnostic or prognostic strategies, helping to identify the one with the highest clinical utility or 'net benefit'. Finally, calibration plots were generated to assess the agreement between observed outcomes and predicted survival probabilities. Survival curves were plotted using the estimated cumulative incidence function from the competing risks framework. Finally, for each model we calculated the proportion of patients with an annual HCC incidence >3%.

Except for descriptive analyses, all other analyses were conducted on imputed data, with imputation performed using a RF-based approach[36] with the R package *missForest*. Finally, as all newly developed models were based on ML approaches (including RFs), explicit feature decorrelation was not performed, as these algorithms are generally robust to correlated predictors.

Statistical analyses were performed using Stata v17.0 (StataCorp, TX, USA), R v4.4.1 (R Foundation for Statistical Computing, Vienna, Austria; using party, partykit,[27] mlr3, missForest, riskRegression and pec packages) and Python 3.7 (DeepHit library)

Results

Selection and baseline characteristics of patients

A total of 5,624 patients with compensated cirrhosis undergoing HCC surveillance and included in the three cohorts were considered (see flowchart, Fig. S3). Among them, 757 were excluded, mostly because of persistent HCV/HBV viral infection during follow-up. The remaining 4,867 patients had either non-viral causes of cirrhosis and/or cured HCV/controlled HBV infections and were included in all subsequent analyses. Their baseline characteristics are displayed in Table 1 as a function of their inclusion in the development (n = 3,251) or validation (n = 1,616) sets. Table S1 also provides the baseline characteristics of patients as a function of their inclusion in the three cohorts.

HCC incidence and survival rates

After a median follow-up of 59.3 (95% CI 57.8; 60.5) months in the development set, 299 (9.2%) patients developed HCC, with a corresponding yearly incidence of 2.06% (95% CI 1.84–2.31). Similarly, 158 (9.8%) HCC cases occurred after 66.5 (95% CI 65.0–67.8) months in the validation set (annual incidence: 2.00% (95% CI 1.72–2.35). The HCC incidence was similar in both sets (subdistribution hazard ratio 0.99, 95% CI 0.82; 1.21, p = 0.915, Fig. S4). During the same timeframe, 396 (12.2%) patients died in the development set. In the validation set, 197 (12.2%) patients died. Overall survival was also similar in both populations (hazard ratio 0.91, 95% CI 0.76–1.07, p = 0.254).

Prognostic model using single decision tree recursive partitioning

For illustrative purposes and to aid in understanding the main interactions between predictors, a recursive partitioning approach was used to build the single decision tree shown in Fig. 1.

Four main predictors were identified by the algorithm, yielding five groups from various combinations of these predictors and demonstrating markedly contrasting risks of HCC, as shown by the corresponding curves at each end node. Low-predicted HCC risk groups had an annual HCC incidence <1%

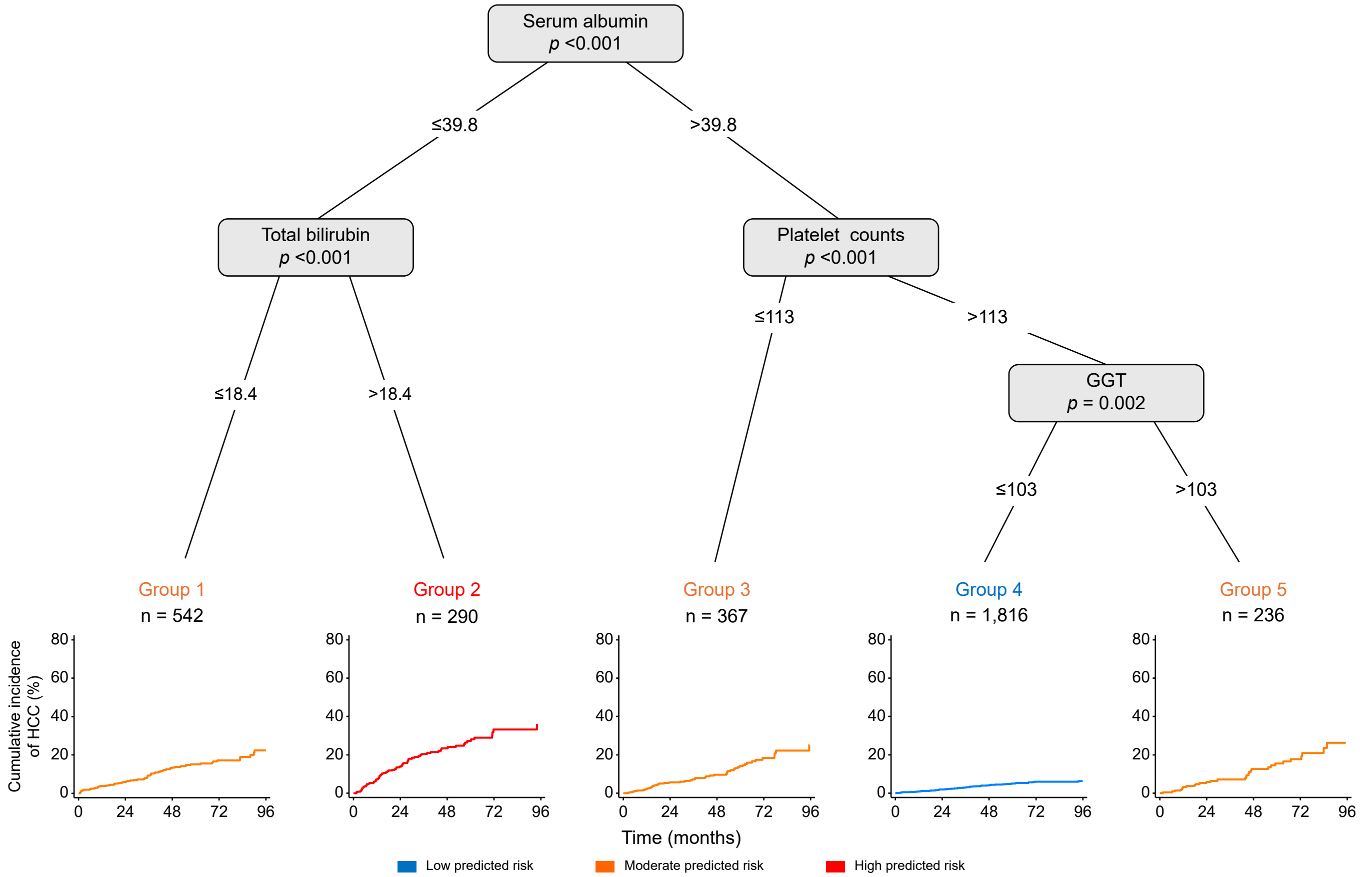

Fig. 1. Decision tree from recursive partitioning analysis of time to HCC occurrence (development cohort; n = 3,251 patients, of whom 299 developed an HCC). Low-predicted HCC risk groups had an annual HCC incidence <1% (n = 1,816, 56%, annual incidence 0.9%); high-predicted HCC risk groups had an annual HCC incidence >5% (n = 290, 9%, annual incidence 6.6%); intermediate-predicted HCC risk groups had an annual HCC incidence between 1% and 5% (n = 1,145, 35%, annual incidence 3.6%). GGT, gamma-glutamyltransferase; HCC, hepatocellular carcinoma.

(n = 1,816, 56%, annual incidence 0.9%); high-predicted HCC risk groups had an annual HCC incidence >5% (n = 290, 9%, annual incidence 6.6%); intermediate-predicted HCC risk groups had an annual HCC incidence between 1% and 5% (n = 1,145, 35%, annual incidence 3.6%).

The most predictive factor in the root node was serum albumin which dichotomized patients into two main subpopulations. Although all patients had Child-Pugh A cirrhosis at inclusion, a slightly impaired albumin level below 39.8 G/L selected patients with a high HCC risk profile, who were furthermore stratified as moderate-(Group 1) or high- (Group 2) risk groups as a function of bilirubin level, the latter group representing an "extreme" phenotype comprising a small subpopulation (n = 290, 9% of the population). Among patients with higher serum albumin levels, surrogate markers of portal hypertension (platelet count) or comorbidities/liver insult (GGT levels) defined several clusters of patients. In particular, patients with high platelet counts and low GGT levels comprised a large group of 1,816 patients (56%, Group 4) with a particularly low HCC incidence. By contrast, patients with either a low platelet count (<113 $x10^3/mm^3$) or high GGT levels (>103 IU/L) identified patients with a moderate risk (Groups 3 and 5).

The clinical and biological characteristics of the five groups generated by decision tree analysis are shown in Table 2. In Group 2, the high-risk group, several additional features were impaired beyond the criteria identified by the decision tree (*e.g.* higher alkaline phosphatase levels and lower prothrombin time) and this group included a higher proportion of patients with metabolic dysfunction- and alcohol-associated liver disease. By contrast, patients in Group 4, representing the lowest HCC risk, were characterized by generally optimal liver parameters and minimal indirect signs of comorbidities, and were enriched for patients with cured or controlled viral-induced cirrhosis.

Prognostic models using random survival forests

Random forests were constructed by aggregating 1,000 decision trees (Fig. S1). Fig. 2 ranks the predictors in order of their relative importance in the RF algorithm, with high importance values indicating the most influential variables predictive of HCC (Fig. 2A) or death without HCC (Fig. 2B). RF approaches confirmed the results of the single decision tree analysis previously described, identifying higher GGT, lower platelet count and albumin levels, but also older age, higher AST and AFP levels as predictive of HCC. Similar to the decision tree analysis, other variables had a weaker influence.

Prognostic models using deep neural networks

We performed five-fold cross-validation on the training set to identify the optimal hyperparameters, selecting those that

Table 2. Comparison of the features of the final groups obtained by decision tree analysis (development cohort; n = 3,210 patients, of whom 294 developed an HCC).

	Group 1	Group 2	Group 3	Group 4	Group 5	*p* value
	n = 542	n = 290	n = 367	n = 1,816	n = 236	
Age	60.9 [54.0–68.9]	59.0 [52.9–67.0]	58.0 [51.8–65.7]	58.0 [51.9–65.8]	57.0 [50.0–63.7]	**<0.001**
Male sex	340 (62.7)	194 (66.9)	233 (63.5)	1,258 (69.3)	171 (72.5)	**0.009**
Platelet count, $10^3/mm^3$	146 [101–197]	94.0 [66.0–135]	89.0 [71.0–102]	186 [156–217]	168 [141–206]	**<0.001**
AST	31.1 [24.0–43.6]	45.0 [34.0–60.0]	32.0 [26.0–44.0]	25.0 [21.0–30.0]	40.5 [30.5–59.5]	**<0.001**
ALT	26.0 [18.0–35.0]	29.0 [22.0–40.0]	29.0 [22.0–43.0]	24.0 [19.0–32.0]	43.0 [29.0–59.0]	**<0.001**
GGT	62.0 [34.0–122]	86.1 [46.0–156]	57.0 [33.0–113]	31.0 [22.0–46.0]	165 [130–259]	**<0.001**
Prothrombin time,	80.0 [72.0–93.0]	68.0 [58.7–75.9]	81.0 [73.0–90.0]	92.6 [86.0–97.3]	88.8 [80.5–96.0]	**<0.001**
Serum albumin, G/L	37.9 [36.0–39.0]	35.4 [32.8–37.5]	42.7 [41.0–44.6]	43.9 [42.3–45.1]	43.1 [41.6–45.0]	**<0.001**
Total bilirubin, μmol/L	10.3 [8.0–14.0]	26.0 [21.7–34.0]	12.5 [9.7–17.0]	9.8 [8.0–11.8]	10.5 [8.0–14.0]	**<0.001**
Alpha-fetoprotein, ng/ml	5.6 [3.50–10.3]	6.3 [4.00–9.8]	6.7 [3.80–11.8]	5.0 [3.00–9.3]	6.5 [4.00–11.6]	**<0.001**
INR	1.17 [1.09–1.25]	1.30 [1.20–1.46]	1.14 [1.10–1.22]	1.07 [1.02–1.12]	1.10 [1.04–1.16]	**<0.001**
Creatinine	70.6 [62.2–83.0]	64.2 [55.1–74.9]	68.7 [60.5–78.8]	72.8 [65.5–81.3]	71.3 [62.7–77.9]	**<0.001**
Glycemia	1.19 [1.01–1.91]	1.31 [1.11–5.1]	1.08 [0.92–1.30]	1.00 [0.93–1.10]	1.19 [1.04–4.00]	**<0.001**
Alkaline phosphatase	103 [81.0–129]	126 [97.0–154]	99.0 [79.1–121]	82.0 [66.0–103]	109 [84.3–140]	**<0.001**
Ferritin	153 [78.0–344]	163 [80.0–358]	225 [125–425]	254 [149–441]	280 [161–548]	**<0.001**
Cirrhosis aetiology						**<0.001**
Cured HCV	367 (67.7)	173 (59.7)	266 (72.5)	1,415 (77.9)	156 (66.1)	
Controlled HBV	60 (11.1)	26 (9.0)	47 (12.8)	311 (17.1)	20 (8.5)	
Alcohol and/or metabolic	115 (21.2)	91 (31.4)	54 (14.7)	90 (5.0)	60 (25.4)	
Cohorts						**<0.001**
CIRRAL	115 (21.2)	91 (31.4)	54 (14.7)	90 (5.0)	60 (25.4)	
CirVir	91 (16.8)	33 (11.4)	93 (25.3)	396 (21.8)	44 (18.6)	
HEPATHER	336 (62.0)	166 (57.2)	220 (59.9)	1,330 (73.2)	132 (55.9)	

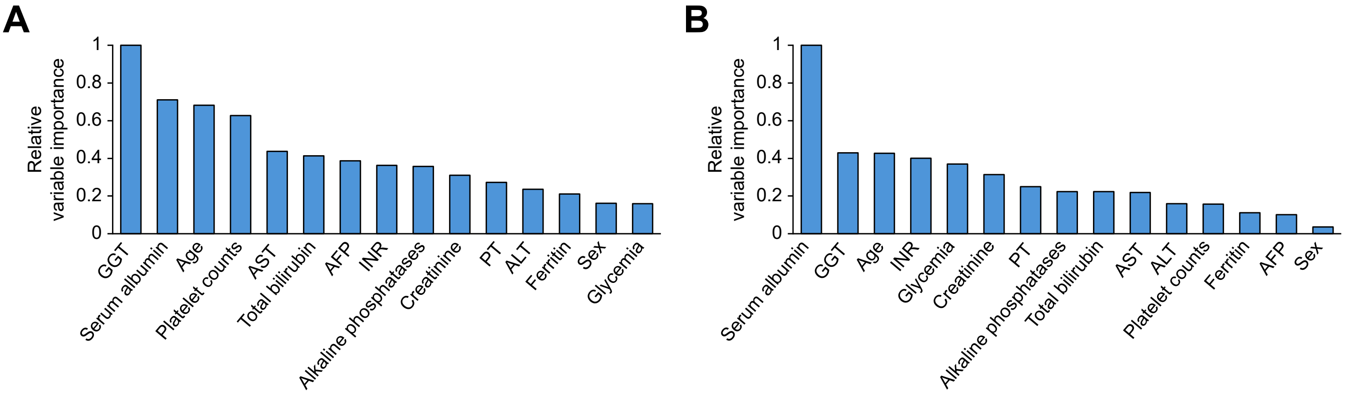

Fig. 2. Variable importance from random survival forest analysis. Variable importance from random survival forest analysis of time to HCC occurrence (A) and HCC-free mortality (B) in the development cohort (n = 3,251 patients, of whom 299 developed an HCC). AFP, alpha-fetoprotein; ALT, alanine aminotransferase; AST, aspartate aminotransferase; GGT, gamma-glutamyltransferase; HCC, hepatocellular carcinoma; PT, prothrombin time.

maximized the average time-dependent AUC (calculated as the mean of three AUCs at 12, 36, and 60 months). The resulting DNN consisted of a shared hidden layer (300 neurons, ELU activation) followed by three cause-specific subnetworks, each with three hidden layers of 300 neurons and ELU activation. The network was trained for 1,000 iterations with a batch size of 128, 40% dropout, and optimized regularization parameters ($\alpha = 0.1$, $\beta = 3$, $\gamma = 0.5$). These optimized hyperparameters were then applied to evaluate model performance in the validation cohort (see Table 3 and S2).

Discriminative performance and calibration in the validation cohort

The discriminative performance of the aMAP and FASTRAK scores, as well as RF and DNN models, at 1, 3 and 5 years was computed and compared in the validation cohort using time-dependent C-indices and Brier scores (Table 3). Comparisons of these metrics did not indicate any substantial advantage of the ML models over traditional scores for HCC prediction at 1-, 3-, or 5-year horizons, a finding further confirmed by decision curve analysis (Fig. 3). Table S2 additionally shows the performances of the different models applied in each of the three cohorts, yielding similar conclusions (Table S2).

To illustrate the clinical value of ML models in discriminating patients with different risk levels, five risk categories were defined based on quintiles of the predicted 3-year HCC risk score from each model. Fig. 4 shows the resulting incidence curves for the cumulative incidence of HCC according to aMAP/FASTRAK scores, decision tree, RF and DNN modeling approaches. Consistent with the computed C-indices and

Table 3. Discrimination performance indices by modeling approach.

	Training set	Validation set
**Time-dependent AUC**		
At 1 year		
aMAP score	0.7380 (0.6558. 0.7921)	0.7285 (0.6488–0.8082)
FASTRAK score	0.7846 (0.7207–0.8265)	0.7207 (0.6385–0.8030)
Decision tree	0.6620 (0.5686. 0.7560)	0.6778 (0.5888–0.7667)
Survival random forest	0.7480 (0.6920–0.8123)	0.7441 (0.6683–0.8199)
Deep neural network	0.7913 (0.7388–0.8437)	0.7525 (0.6731–0.8320)
At 3 years		
aMAP score	0.7491 (0.7133–0.7918)	0.6953 (0.6400–0.7506)
FASTRAK score	0.7464 (0.6931–0.7956)	0.7154 (0.6630–0.7678)
Decision tree	0.6746 (0.6211–0.7311)	0.6682 (0.6143–0.7220)
Survival random forest	0.7364 (0.6832–0.7851)	0.7263 (0.6778–0.7749)
Deep neural network	0.7851 (0.7526–0.8175)	0.7245 (0.6727–0.7764)
At 5 years		
aMAP score	0.7021 (0.6599–0.7322)	0.6755 (0.6273–0.7237)
FASTRAK score	0.7062 (0.6585–0.7400)	0.6893 (0.6418–0.7368)
Decision tree	0.6501 (0.5792–0.6988)	0.6499 (0.6029–0.6969)
Survival random forest	0.7040 (0.6598–0.7481)	0.6832 (0.6349–0.7315)
Deep neural network	0.7369 (0.7052–0.7686)	0.6986 (0.6496–0.7476)
**Brier score**		
At 1 year		
aMAP score	0.022 (0.015–0.030)	0.021 (0.015–0.028)
FASTRAK score	0.022 (0.015–0.030)	0.021 (0.015–0.028)
Decision tree	0.022 (0.015–0.030)	0.021 (0.015–0.028)
Survival random forest	0.022 (0.014–0.030)	0.021 (0.015–0.028)
Deep neural network	0.021 (0.016–0.025)	0.022 (0.015–0.028)
At 3 years		
aMAP score	0.055 (0.050–0.065)	0.062 (0.051–0.073)
FASTRAK score	0.055 (0.050–0.064)	0.060 (0.050–0.071)
Decision tree	0.058 (0.052–0.068)	0.062 (0.051–0.073)
Survival random forest	0.055 (0.050–0.064)	0.061 (0.051–0.072)
Deep neural network	0.055 (0.048–0.061)	0.062 (0.052–0.071)
At 5 years		
aMAP score	0.086 (0.079–0.098)	0.087 (0.075–0.100)
FASTRAK score	0.085 (0.079–0.096)	0.086 (0.074–0.098)
Decision tree	0.090 (0.083–0.101)	0.087 (0.075–0.099)
Survival random forest	0.085 (0.079–0.097)	0.087 (0.075–0.099)
Deep neural network	0.087 (0.080–0.094)	0.090 (0.079–0.100)

Brier scores, a clearly graded relation between predicted risk and observed HCC occurrence was apparent for both Cox models (aMAP/FASTRAK scores) and RF or DNN. Finally, calibration curves for the validation set showed that both ML approaches and traditional models maintained similar alignment between predicted and observed risks (Fig. 5).

Finally, for each model, we calculated the proportion of patients with an annual HCC incidence >3%. ML models identified higher proportions of patients with an annual HCC incidence >3%: ST 1,435/3,251 (44%); DNN 1,202/3,251 (37%); RF 975/3,251 (30%) *vs*. aMAP 1,179/3,251 (36%); FASTRAK 941/3,251 (29%).

Discussion

The findings from the present report provide a comprehensive evaluation of both traditional and ML-based approaches in the context of HCC surveillance programmes. Our findings offer valuable insights into the use of ML methodologies for HCC risk prediction, demonstrating the potential and limitations of such models within clinical practice. Importantly, although the RF models revealed interactions between variables and identified patient clusters with differing risk levels – features not captured by traditional scoring systems – the overall predictive performance was comparable.

One of the key contributions of this study is the ML models' ability to stratify patients into specific risk subpopulations and highlight individuals with particularly high-risk profiles. The identification of clinical predictors such as GGT levels, platelet counts, and albumin through the ST (Fig. 1) and RF (Fig. 2) models echoes known risk factors, suggesting that ML models can validate and potentially reinforce the clinical importance of variables well-documented in HCC risk literature.[37] Algorithms using ML approaches applied to the French cohorts also provided additional insights compared with traditional logistic regression models. First, by demonstrating the interactions between variables, they help identify clusters of patients defined by different HCC risk levels. For instance, the recursive partitioning approach (Fig. 1, Table 2) shows that in this population, slightly decreased albumin levels in otherwise compensated patients, combined with higher bilirubin, define a small subset of patients (9%) with an annual HCC incidence >5%. This observation suggests that even minimal impairment of hepatic function has a major impact on the risk of HCC. Moreover, higher GGT levels – likely indicative of associated comorbidities – appear to further refine individual risk, particularly by identifying patients with otherwise perfectly compensated liver function who have an intermediate HCC risk of >3%. Beyond the notably illustrative aspect of these ST analyses for clinicians, the RF model, constructed using an ensemble of 1,000 decision trees, corroborated the predictors identified by the ST model and reinforced their importance (Fig. 2). Interestingly, epidemiological factors such as older age and non-specific biomarkers like higher GGT levels were among the most influential predictors, alongside parameters estimating liver function, whereas non-hepatic markers appeared to be less influential. Finally, the RF model also highlighted the complex interactions among variables, a strength of ML methods that may be overlooked in traditional regression-based approaches.

These observations could be pivotal for personalized clinical decision-making, allowing targeted resource allocation and enhanced monitoring strategies for patients identified as being at higher HCC risk. For instance, it has been shown that HCC surveillance based on liver MRI examination would be cost-effective for very-early HCC (BCLC 0) detection as soon as annual HCC incidence surpasses 3%.[4] This hypothesis is currently being tested in the setting of a French randomized trial (FASTRAK trial, NCT NCT05095714), in which the selection of patients is based on the FASTRAK scoring system used as comparator in the present work, which has been shown to enable the identification of roughly 30% of patients with an annual HCC incidence >3%.[14] This finding was confirmed in the present analyses conducted in larger cohorts. For instance, the present ST model showed that this proportion could be increased to 44% (intermediate- and high-predicted HCC risk groups, Fig. 1). The application of such a scoring system for allocation into MRI surveillance programmes would increase the detection of early-stage tumours eligible for curative treatments – such as surgery, ablation, or transplantation – a strategy highlighted by Europe's Beating Cancer Plan as the most effective measure to improve the poor prognosis of liver cancer.[38,39]

Despite their added sophistication, ML models offered no substantial discriminative advantage over established scoring

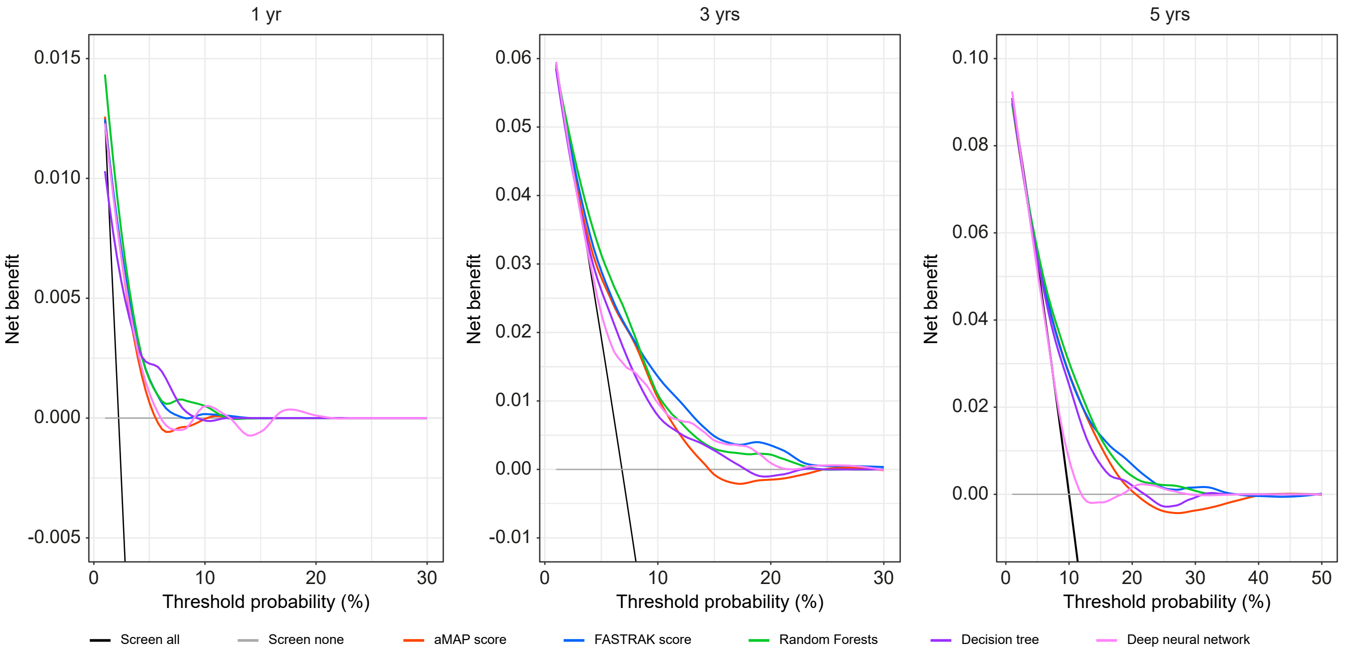

Fig. 3. Decision curves display the net benefit between surveillance benefits and surveillance harms. Net benefit in screened patients is calculated across a range of HCC risk thresholds (defined as the minimum probability of disease at which biannual screening would be warranted), as the proportion of patients with true positive results minus the proportion with false-positives multiplied by the odds at the threshold probability (HCC risk/1 – HCC risk). The net benefits of the risk prediction models were compared with those from two different reference strategies of screening all patients or none. Risk models with higher discriminative power will provide higher net benefit, as evidenced by the highest plotted decision curves. HCC, hepatocellular carcinoma.

systems (Table 3 and S2, Fig. 4), even when flexible and highly complex approaches, such as DNNs, were included.

The extent to which ML models may help in detecting subtle patterns that emerge over longer follow-up periods remains to be explored. Calibration curves for the validation set showed that both ML approaches and traditional models maintained similar alignment between predicted and observed risks (Fig. 5).

Of note, our previously published FASTRAK score, initially developed in a subgroup of the present population,[4] was validated following the incorporation of 1,864 (38%) additional patients. This suggests that while ML techniques can enrich data-driven risk stratification, their incremental benefit over conventional statistical methods may be context-dependent. The results indicate that existing models, such as the previously published aMAP[31] or FASTRAK scores,[4] are robust for immediate- and medium-term HCC risk prediction, while ML models might be better suited for scenarios in which predictive risk factors or biomarkers only relevant in highly specific subgroups can be identified. For example, genetic variants (*PNPLA3, TM6SF2*, and others) associated with HCC identified through large-scale pangenomic studies have shown only modest improvements in risk stratification when integrated into regression models in longitudinal cohorts under surveillance.[40] These findings suggest that such genetic biomarkers may have a significant impact only in specific patient subgroups, which are not adequately captured by traditional statistical methods. The potential of newly identified risk factors to enhance HCC risk stratification using ML approaches is highly anticipated.[13]

Despite the promise of ML models, there are several considerations to note. The computational complexity and interpretability of ML models, especially when compared to simpler scoring systems, may pose challenges in clinical adoption. Traditional models benefit from their ease of use and clear clinical interpretation, which remain critical for broad implementation in clinical practice. In this context, the added complexity might not justify widespread substitution of established models without further validation across diverse populations and settings, particularly outside France. However, the ML models' potential to uncover nuanced interactions might serve as a complementary tool, particularly in the presence of heterogeneous patient populations. It should also be noted that our analysis was limited to a selected set of ML approaches adapted for competing-risks survival analysis, and that other existing or future methods may yield improved performance. Finally, our findings are specific to the present dataset and the predictors available, and may not be generalizable to other populations or clinical settings. Future research should explore the integration of ML-based risk stratification tools into clinical workflows, potentially as complementary tools to traditional scores rather than replacements.

In conclusion, our study highlights that ML models can reveal interactions between variables, identify patient clusters with distinct risk profiles, and detect extreme phenotypes associated with particularly high HCC risk. However, when only combining clinical and biological routine parameters, they do not yet demonstrate a significant performance advantage

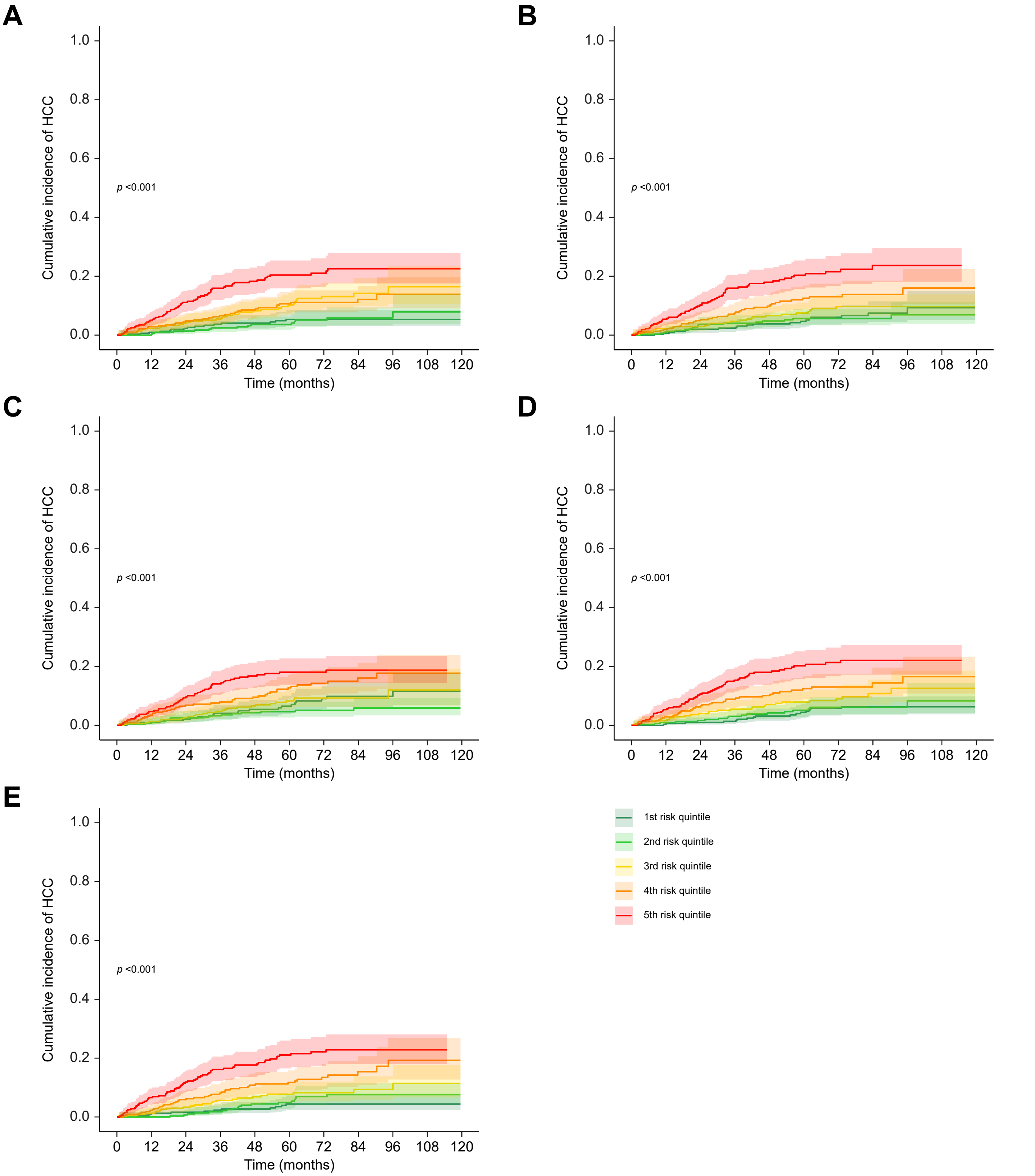

Fig. 4. Cumulative incidence curves of HCC by 3-year quintiles of predicted risk according to different modelling approaches using the whole follow-up of the validation cohort (n = 1,616 patients, of whom 158 developed an HCC). (A) aMAP score. (B) FASTRAK score. (C) Decision tree. (D) Random survival forest. (E) Deep neural network. Levels of significance: *p* <0.05) (Gray's test). HCC, hepatocellular carcinoma.

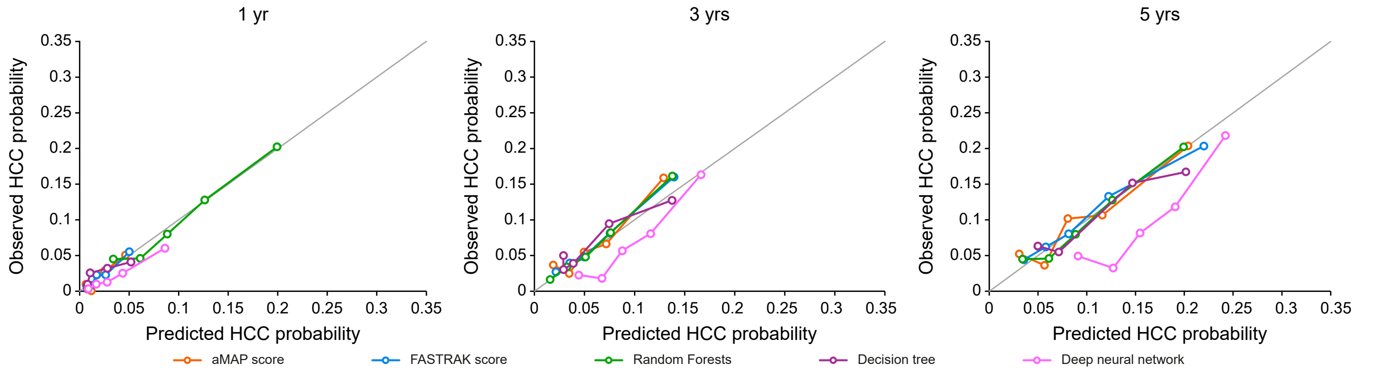

Fig. 5. Calibration plots of machine learning and traditional models in the validation population (n = 1,616 patients, of whom 158 developed an HCC). HCC, hepatocellular carcinoma.

over traditional models. Their enrichment by newly identified risk factors derived from circulating biobanks or imaging examinations is highly anticipated.[40,41] The development and application of these models will need to balance predictive power, clinical utility, and interpretability to optimize patient care in HCC surveillance programmes.

Affiliations

[1]*AP-HP, Hôpitaux Universitaires Paris Seine Saint-Denis, APHP, Liver Unit, Université Sorbonne Paris Nord, F-93000 Bobigny, Inserm, UMR-1138 "Functional Genomics of Solid Tumors", Centre de recherche des Cordeliers, Université de Paris, Paris, France;* [2]*Univ Paris Est Créteil, INSERM, IMRB, Equipe CEpiA (Clinical Epidemiology and Ageing), Unité de Recherche Clinique (URC Mondor), Service de Santé Publique, Assistance Publique Hôpitaux de Paris (AP-HP), Hôpitaux Universitaires Henri Mondor, F-94000, Créteil, France;* [3]*Université de Paris, département d'hépatologie/Addictologie, Hôpital Cochin, APHP, Paris, France;* [4]*Clinical Research Department, ANRS | Emerging Infectious Diseases, Paris, France;* [5]*SBIM, APHP, Hôpital Saint-Louis, Paris, Inserm, UMR-1153, ECSTRA Team, Paris, France;* [6]*Université Sorbonne Paris Nord, Laboratory for Vascular Translational Science, LVTS, INSERM, UMR 1148, F-93430, Villetaneuse, Université de Paris, F-75018, Paris, AP-HP, HUPSSD, Hôpital Avicenne, Laboratoire de Biochimie, F-93000, Bobigny, France;* [7]*Sorbonne Université, Inserm, Institut Pierre Louis d'Epidémiologie et de Santé Publique, AP-HP, Hôpital Saint-Antoine, Unité de Santé Publique, Paris, France*

Abbreviations

AFP, alpha-fetoprotein; AST, aspartate aminotransferase; DNN, deep neuronal network; GGT, gamma-glutamyltransferase; HCC, hepatocellular carcinoma; ML, machine learning; ST, single tree; RF, random (survival) forest.

Financial support

The promoters of the three prospective cohorts were the Assistance Publique des Hôpitaux de Paris (APHP) for CIRRAL and ANRS for CirVir and Hepather. The cohorts were funded by 1) 1) the National Agency for Research on HIV and Hepatitis (ANRS) for CirVir and Hepather, and 2) CIRRAL: the French National Institut of Cancer (INCa), the French Association for Research in Cancer and the ANRS (PAIR CHC 2009). Pierre Nahon's research is funded in part by the European Union (GENIAL, Grant agreement ID: 101096312), French Agence Nationale de la Recherche (France 2030 DELIVER ANR-21-RHUS-0001) and by France 2030 RHU LIVER-TRACK (ANR-23-RHUS-0014).

Conflicts of interest

Pr Nahon has received honoraria from and/or consults for AstraZeneca, Bristol-Myers Squibb, Eisai, and Roche. He received research grants from AstraZeneca, Bristol-Myers Squibb and Eisai. Pr Ganne-Carrié consults for and/or received personal fees from Abbvie, Bayer, Gilead, Ipsen, and Shionogi, outside the submitted work.
Please refer to the accompanying ICMJE disclosure forms for further details.

Authors' contributions

Drs Nahon and Audureau had full access to all data in the study and take responsibility for data integrity and the accuracy of data analysis. *Study concept and design:* Nahon, Audureau. *Acquisition of data:* Nahon, Parlati, Lusivika Nzinga, Carrat, Ganne-Carrié, N'Kontchou, Chaffaut, Bamba-Funck, Sutton. *Analysis and interpretation of data:* Nahon, Layese, Natella, Audureau. *Drafting of the manuscript:* Nahon, Layese, Audureau. *Critical revision of the manuscript for important intellectual content:* all authors. *Statistical analyses:* Layese, Audureau. *Obtained* funding*:* Nahon, Ganne-Carrié, Parlati, Carrat. *Administrative, technical and material* support*:* Nahon, Saidi, Layese, Natella, Audureau. *Study supervision:* Nahon, Audureau.

Data availability

Data can be made available upon reasonable request.

Role of the sponsor

The funding sponsors had no role in the design and conduct of the study; the collection, management, analysis or interpretation of the data; or the preparation, review or approval of the manuscript.

Declaration of generative AI and AI-assisted technologies in the writing process

During the preparation of this work the author(s) used ChatGPT in order to check for grammar/spelling. ChatGPT was also partially used to design the Graphical abstract. After using this tool, the authors reviewed and edited the content as needed and take full responsibility for the content of the publication.

Supplementary data

Supplementary data to this article can be found online at https://doi.org/10.1016/j.jhepr.2026.101740.

References

[1] European Association for the Study of the Liver. EASL Clinical Practice Guidelines on the management of hepatocellular carcinoma. J Hepatol 2025;82:315–374.
[2] Ronot M, Nahon P, Rimola J. Screening of liver cancer with abbreviated MRI. Hepatology 2023;78:670–686.
[3] Singal AG, Sanduzzi-Zamparelli M, Nahon P, et al. International Liver Cancer Association (ILCA) white paper on hepatocellular carcinoma risk stratification and surveillance. J Hepatol 2023;79:226–239.
[4] Nahon P, Najean M, Layese R, et al. Early hepatocellular carcinoma detection using magnetic resonance imaging is cost-effective in high-risk patients with cirrhosis. JHEP Rep 2022;4:100390.
[5] Kanwal F, Khaderi S, Singal AG, et al. Risk factors for HCC in contemporary cohorts of patients with cirrhosis. Hepatology 2023;77:997–1005.

[6] Ganne-Carrie N, Nahon P. Hepatocellular carcinoma in the setting of alcohol-related liver disease. J Hepatol 2019;70:284–293.
[7] Ganne-Carrie N, Nahon P. Differences between hepatocellular carcinoma caused by alcohol and other aetiologies. J Hepatol 2025;82:909–917.
[8] Kanwal F, Kramer JR, Mapakshi S, et al. Risk of hepatocellular cancer in patients with non-alcoholic fatty liver disease. Gastroenterology 2018;155:1828–1837 e1822.
[9] Nahon P, Vo Quang E, Ganne-Carrie N. Stratification of hepatocellular carcinoma risk following HCV eradication or HBV control. J Clin Med 2021;10(2):353.
[10] Nahon P, Layese R, Bourcier V, et al. Incidence of hepatocellular carcinoma after direct antiviral therapy for HCV in patients with cirrhosis included in surveillance programs. Gastroenterology 2018;155:1436–1450 e1436.
[11] Brichler S, Nahon P, Zoulim F, et al. Non-virological factors are drivers of hepatocellular carcinoma in virosuppressed hepatitis B cirrhosis: results of ANRS CO12 CirVir cohort. J viral Hepat 2019;26:384–396.
[12] Ganne-Carrie N, Chaffaut C, Bourcier V, et al. Estimate of hepatocellular carcinoma incidence in patients with alcoholic cirrhosis. J Hepatol 2018;69:1274–1283.
[13] Innes H, Nahon P. Statistical perspectives on using hepatocellular carcinoma risk models to inform surveillance decisions. J Hepatol 2023;79:1332–1337.
[14] Nahon P, Ronot M, Sutter O, et al. Study protocol for FASTRAK: a randomised controlled trial evaluating the cost impact and effectiveness of FAST-MRI for HCC suRveillance in pAtients with high risK of liver cancer. BMJ Open 2024;14:e083701.
[15] Ngiam KY, Khor IW. Big data and machine learning algorithms for health-care delivery. Lancet Oncol 2019;20:e262–e273.
[16] Trinchet JC, Chaffaut C, Bourcier V, et al. Ultrasonographic surveillance of hepatocellular carcinoma in cirrhosis: a randomized trial comparing 3- and 6-month periodicities. Hepatology 2011;54:1987–1997.
[17] Trinchet JC, Bourcier V, Chaffaut C, et al. Complications and competing risks of death in compensated viral cirrhosis (ANRS CO12 CirVir prospective cohort). Hepatology 2015;62:737–750.
[18] Carrat F, Fontaine H, Dorival C, et al. Clinical outcomes in patients with chronic hepatitis C after direct-acting antiviral treatment: a prospective cohort study. Lancet 2019;393:1453–1464.
[19] Bruix J, Sherman M. Management of hepatocellular carcinoma. Hepatology 2005;42:1208–1236.
[20] Bruix J, Sherman M. Management of hepatocellular carcinoma: an update. Hepatology 2011;53:1020–1022.
[21] EASL clinical practice guidelines: management of hepatocellular carcinoma. J Hepatol 2018;69:182–236.
[22] Thabut D, Bureau C, Layese R, et al. Validation of baveno VI criteria for screening and surveillance of esophageal varices in patients with compensated cirrhosis and a sustained response to antiviral therapy. Gastroenterology 2019;156:997–1009 e1005.
[23] Nahon P, Lescat M, Layese R, et al. Bacterial infection in compensated viral cirrhosis impairs 5-year survival (ANRS CO12 CirVir prospective cohort). Gut 2017;66:330–341.
[24] Allaire M, Nahon P, Layese R, et al. Extrahepatic cancers are the leading cause of death in patients achieving hepatitis B virus control or hepatitis C virus eradication. Hepatology 2018;68:1245–1259.
[25] Cacoub P, Nahon P, Layese R, et al. Prognostic value of viral eradication for major adverse cardiovascular events in hepatitis C cirrhotic patients. Am Heart J 2018;198:4–17.
[26] Audureau E, Carrat F, Layese R, et al. Personalized surveillance for hepatocellular carcinoma in cirrhosis - using machine learning adapted to HCV status. J Hepatol 2020;73:1434–1445.
[27] Torsten Hothorn KH, Zeileis Achim. Unbiased recursive partitioning: a conditional inference framework. J Comput Graphical Stat 2006;15:651–674.
[28] Ishwaran H, Kogalur UB. Consistency of random survival forests. Stat Probab Lett 2010;80:1056–1064.
[29] Wright M. Feature selection. In: Bischl B, Sonabend R, Kotthoff L, Lang M, editors. Applied machine learning using mlr3 in R. CRC Press; 2024.
[30] Lee C, Yoon J, Schaar MV. Dynamic-DeepHit: a deep learning approach for dynamic survival analysis with competing risks based on longitudinal data. IEEE Trans Biomed Eng 2020;67:122–133.
[31] Fan R, Papatheodoridis G, Sun J, et al. aMAP risk score predicts hepatocellular carcinoma development in patients with chronic hepatitis. J Hepatol 2020;73:1368–1378.
[32] Kamarudin AN, Cox T, Kolamunnage-Dona R. Time-dependent ROC curve analysis in medical research: current methods and applications. BMC Med Res Methodol 2017;17:53.
[33] Fe H. Regression modeling strategies: with applications to linear models. Logistic regression, and survival analysis. New York, NY: Springer; 2001.
[34] Vickers AJ, Elkin EB. Decision curve analysis: a novel method for evaluating prediction models. Med Decis Making 2006;26:565–574.
[35] Vickers AJ, Van Calster B, Steyerberg EW. Net benefit approaches to the evaluation of prediction models, molecular markers, and diagnostic tests. BMJ 2016;352:i6.
[36] Stekhoven DJ, Buhlmann P. MissForest–non-parametric missing value imputation for mixed-type data. Bioinformatics 2012;28:112–118.
[37] Singal AG, Lampertico P, Nahon P. Epidemiology and surveillance for hepatocellular carcinoma: new trends. J Hepatol 2020;72:250–261.
[38] Lawler M, Davies L, Oberst S, et al. European groundshot-addressing Europe's cancer research challenges: a Lancet oncology commission. Lancet Oncol 2023;24:e11–e56.
[39] Kondili LA, Lazarus JV, Jepsen P, et al. Inequities in primary liver cancer in Europe: the state of play. J Hepatol 2024;80:645–660.
[40] Nahon P, Bamba-Funck J, Layese R, et al. Integrating genetic variants into clinical models for hepatocellular carcinoma risk stratification in cirrhosis. J Hepatol 2023;78:584–595.
[41] Dana J, Meyer A, Paisant A, et al. Improving risk stratification and detection of early HCC using ultrasound-based deep learning models. JHEP Rep 2025;7:101510.

Keywords: cirrhosis; HCC risk stratification; machine learning; surveillance; cost-effectiveness.
Received 24 July 2025; received in revised form 8 January 2026; accepted 13 January 2026; Available online 3 February 2026

Journal of Hepatology, Volume *8*

Supplemental information

Comparative evaluation of regression and machine-learning models for hepatocellular carcinoma risk stratification across diverse aetiologies

Pierre Nahon, Richard Layese, Pierre-André Natella, Lucia Parlati, Tounes Saidi, Nathalie Ganne-Carrié, Gisèle N'Kontchou, Cendrine Chaffaut, Jean-Charles Nault, Jessica Bamba-Funck, Angela Sutton, Clovis Lusivika Nzinga, Fabrice Carrat, Etienne Audureau, and for the ANRS CO12 CirVir, ANRS CO22 Hepather, and CIRRAL groups

Comparative evaluation of regression and machine-learning models for hepatocellular carcinoma risk stratification across diverse aetiologies

Pierre Nahon, Richard Layese, Pierre-André Natella, Lucia Parlati, Tounes Saidi, Nathalie Ganne-Carrié, Gisèle N'Kontchou, Cendrine Chaffaut, Jean-Charles Nault, Jessica Bamba-Funck, Angela Sutton, Clovis Lusivika Nzinga, Fabrice Carrat, Etienne Audureau, for the ANRS CO12 CirVir, ANRS CO22 Hepather, and CIRRAL groups

Table of contents

List of investigators……………………………………………………………..………2

Supplementary figures……………………………………………………………..…...5

Supplementary tables…………………………………………………….……….…...9

LIST OF INVESTIGATORS

ANRS CO12 CirVir group:
Pierre Nahon[1], Tarik Asselah[2], Dominique Guyader[3], Stanislas Pol[4], Hélène Fontaine[4], Georges-Philippe Pageaux[5], Victor De Lédinghen[6], Denis Ouzan[7], Fabien Zoulim[8], Dominique Roulot[9], Albert Tran[10], Jean-Pierre Bronowicki[11], Thomas Decaensi[12], Ghassan Riachi[13], Paul Calès[14], Jean-Marie Péron[15], Laurent Alric[16], Marc Bourlière[17], Philippe Mathurin[18], Sebastien Dharancy[18], Jean-Frédéric Blanc[19], Armand Abergel[20], Olivier Chazouillères[21], Ariane Mallat[22], Jean-Didier Grangé[23], Pierre Attali[24], Louis d'Alteroche[25], Claire Wartelle[26], Thông Dao[27], Dominique Thabut[28], Christophe Pilette[29], Christine Silvain[30], Christos Christidis[31], Eric Nguyen-Khac[32], Brigitte Bernard-Chabert[33], Sophie Hillaire[34], Vincent Di Martino[35].
[1]AP-HP, Hôpital Avicenne, Service d'Hépatologie, Bobigny, Université Paris 13, Bobigny et INSERM U1162, Université Paris 5, Paris; [2]AP-HP, Hôpital Beaujon, Service d'Hépatologie, and University Paris Diderot, Sorbonne Paris Cité, CRI, UMR 1149; [3]CHU Pontchaillou, Service d'Hépatologie, Rennes; [4]AP-HP, Hôpital Cochin, Département d'Hépatologie et INSERM UMS20 et U1223, Institut Pasteur, Université Paris Descartes, Paris; [5]Hôpital Saint Eloi, Service d'Hépatologie, Montpellier; [6]Hôpital Haut-Lévêque, Service d'Hépatologie, Bordeaux; [7]Institut Arnaud Tzanck, Service d'Hépatologie, St Laurent du Var; [8]Hôpital Hôtel Dieu, Service d'Hépatologie, Lyon; [9]AP-HP, Hôpital Avicenne, Service d'Hépatologie, Bobigny; [10]CHU de Nice, Service d'Hépatologie, et INSERM U1065, Université de Nice-Sophia-Antipolis, Nice; [11]Hôpital Brabois, Service d'Hépatologie, Vandoeuvre-les-Nancy; [12]Hôpital Michallon, Service d'Hépatologie, Grenoble; [13]Hôpital Charles-Nicolle, Service d'Hépatologie, Rouen; [14]CHU d'Angers, Service d'Hépatologie, Angers; [15]Hôpital Purpan, Service d'Hépatologie, Toulouse; [16]CHU Toulouse, Service de Médecine Interne-Pôle Digestif UMR 152, Toulouse; [17]Hôpital Saint Joseph, Service d'Hépatologie, Marseille; [18]Hôpital Claude Huriez, Service d'Hépatologie, Lille; [19]Hôpital St André, Service d'Hépatologie, Bordeaux; [20]Hôpital Hôtel Dieu, Service d'Hépatologie, Clermont-Ferrand; [21]AP-HP, Hôpital Saint-Antoine, Service d'Hépatologie, Paris; [22]AP-HP, Hôpital Henri Mondor, Service d'Hépatologie, Créteil; [23]AP-HP, Hôpital Tenon, Service d'Hépatologie, Paris; [24]AP-HP, Hôpital Paul Brousse, Service d'Hépatologie, Villejuif; [25]Hôpital Trousseau, Unité d'Hépatologie, CHRU de Tours; [26]Hôpital d'Aix-En-Provence, Service d'Hépatologie, Aix-En-Provence; [27]Hôpital de la Côte de Nacre, Service d'Hépatologie, Caen; [28]AP-HP, Groupe Hospitalier de La Pitié-Salpêtrière, Service d'Hépatologie, Paris; [29]CHU Le Mans, Service d'Hépatologie, Le Mans; [30]CHU de Poitiers, Service d'Hépatologie, Poitiers; [31]Institut Mutualiste Montsouris, Service d'Hépatologie, Paris; [32]Hôpital Amiens Nord, Service d'Hépatologie, Amiens; [33]Hôpital Robert Debré, Service d'Hépatologie, Reims; [34]Hôpital Foch, Service d'Hépatologie, Suresnes; [35]Hôpital Jean Minjoz, Service d'Hépatologie, Besançon. FRANCE.
Funding/Support: This study was sponsored by ANRS (France REcherche Nord & sud SIDA-HIV Hépatites: FRENSH).
This work is dedicated to the memory of Professor Jean-Claude Trinchet.

ANRS CO22 Hepather group:
Funding
INSERM-ANRS (France REcherche Nord&sud Sida-vih Hepatites), ANR (Agence Nationale de la Recherche), DGS (Direction Générale de la Santé) and MSD, Janssen, Gilead, Abbvie, BMS, Roche.
ANRS-AFEF Hepather Study group
Delphine Bonnet, Virginie Payssan-Sicart, Chloe Pomes (CHU Purpan, Toulouse, France), François Bailly, Marjolaine Beaudoin, Dominique Giboz, Kerstin Hartig-Lavie, Marianne Maynard (Hospices Civils de Lyon, Lyon, France), Eric Billaud, David Boutoille, Morane Cavellec, Marjorie Cheraud-Carpentier (Hôpital Hôtel-Dieu, Nantes, France), Isabelle Hubert, Jaouad Benhida, Adrien Lannes, Françoise Lunel, Frédéric Oberti (CHU Angers, Angers, France), Nathalie Boyer, Nathalie Giuily, Corinne Castelnau, Giovanna Scoazec (Hôpital Beaujon, Clichy, France), Aziza

Chibah, Sylvie Keser, Karim Bonardi, Anaïs Vallet-Pichard, Philippe Sogni (Hôpital Cochin, Paris, France), Juliette Foucher, Jean-Baptiste Hiriart, Amy Wilson, Sarah Shili, Faiza Chermak (Hôpital Haut-Lévêque, Pessac, Bordeaux, France), Christelle Ansaldi, Nisserine Ben Amara, Laëtitia Chouquet, Emilie De Luca, Valérie Oules (Hôpital Saint Joseph, Marseille, France), Rodolphe Anty, Eve Gelsi, Régine Truchi (CHU de Nice, Nice, France), Elena Luckina, Nadia Messaoudi, Joseph Moussali (Hôpital de la Pitié Salptétrière, Paris, France), Barbara De Dieuleveult, Damien Labarriere, Pascal Poter, Si Nafa Si Ahmed (CHR La Source, Orléans, France), Véronique Grando-Lemaire, Pierre Nahon, Valérie Bourcier, Séverine Brulé, Thomas Stalhberger (Hôpital Avicenne, Bobigny, France), Caroline Jezequel, Audrey Brener, Anne Laligant, Aline Rabot, Isabelle Renard (CHU Rennes, Rennes, France), Thomas F. Baumert, Michel Dofföel, Catherine Mutter, Pauline Simo-Noumbissie, Esma Razi (Hôpitaux Universitaires de Strasbourg, Strasbourg, France), Hélène Barraud, Mouni Bensenane, Abdelbasset Nani, Sarah Hassani-Nani, Marie-Albertine Bernard (CHU de Nancy, Nancy, France), Georges-Philippe Pageaux, Michael Bismuth, Ludovic Caillo, Stéphanie Faure, Marie Pierre Ripault (Hôpital Saint Eloi, Montpellier, France), Christophe Bureau, Jean Marie Peron, Marie Angèle Robic, Léa Tarallo (CHU Purpan, Toulouse, France), Marine Faure, Bruno Froissart, Marie-Noelle Hilleret, Jean-Pierre Zarski (CHU de Grenoble, Grenoble, France), Odile Goria, Victorien Grard, Hélène Montialoux (CHU Charles Nicolle, Rouen, France), Muriel François, Christian Ouedraogo, Christelle Pauleau, Anne Varault (Hôpital Henri Mondor, Créteil, France), Tony Andreani, Bénédicte Angoulevant, Azeline Chevance, Lawrence Serfaty (Hôpital Saint-Antoine, Paris, France), Teresa Antonini, Audrey Coilly, Jean-Charles Duclos Vallée, Mariagrazia Tateo (Hôpital Paul Brousse, Villejuif, France), Corinne Bonny, Chanteranne Brigitte, Géraldine Lamblin, Léon Muti (Hôpital Estaing, Clermont-Ferrand, France), Abdenour Babouri, Virginie Filipe (Centre Hospitalier Régional, Metz, France), Camille Barrault, Laurent Costes, Hervé Hagège, Soraya Merbah (Centre Hospitalier Intercommunal, Créteil, France), Paul Carrier, Maryline Debette-Gratien, Jérémie Jacques (CHU Limoges, Limoges, France), Guillaume Lassailly, Florent Artu, Valérie Canva, Sébastien Dharancy, Alexandre Louvet (CHRU Claude Huriez, Lille, France), Marianne Latournerie, Marc Bardou, Thomas Mouillot (Dijon University Hospital, Dijon, France), Yannick Bacq, Didier Barbereau, Charlotte Nicolas (CHU Trousseau, 37044 Tours, France), Caroline Chevalier, Isabelle Archambeaud, Sarah Habes (CHU de Nantes, Nantes, France), Nisserine Ben Amara, Danièle Botta-Fridlund, (CHU Timone, Marseille, France), Eric Saillard, Marie-Josée Lafrance, (CHU de Pointe-à-Pitre, Pointe-à-Pitre, Guadeloupe).

Scientific Committee:

- Voting members:

Marc Bourlière (Hôpital St Joseph, Marseille), Patrice Cacoub (Hôpital Pitié salpêtrière, Paris, France, Fabrice Carrat (Scientific Coordinator, Hôpital Saint-Antoine, Paris, France), Patrizia Carrieri (INSERM U912, Marseille, France), Elisabeth Delarocque-Astagneau (Inserm UMR1181, Paris), Victor De Ledinghen (Hôpital Haut-Lévêque, Pessac, Bordeaux, France), Céline Dorival (UPMC & INSERM U1136, Paris, France), Jean Dubuisson (Inserm U1019, Lille, France), Hélène Fontaine (Hôpital Cochin, Paris, France), Chantal Housset (Inserm UMR-S938 1 IFR65, Paris), Dominique Larrey (Hôpital Saint Eloi, Montpellier, France), Patrick Marcellin (Hôpital Beaujon, Clichy, France), Philippe Mathurin (CHRU Claude Huriez, Lille, France), Pierre Nahon (Hôpital Avicenne, Bobigny, France), Georges-Philippe Pageaux (Hôpital Saint Eloi, Montpellier, France), Jean-Michel Pawlotsky (Hôpital Henri Mondor, Créteil, France), Ventzislava Petrov-Sanchez (ANRS, Paris, France), Stanislas Pol (Principal Investigator, Hôpital Cochin, Paris, France), Sophie Vaux (Agence Nationale de Santé Publique, Saint Maurice, France), Linda Wittkop (ISPED-INSERM U897, Bordeaux, France), Yazdan Yazdanpanah (Hôpital Bichat Claude Bernard, Paris, France), Jean-Pierre Zarski (CHU de Grenoble, Grenoble, France), Fabien Zoulim (Hospices Civils de Lyon, Lyon, France), Jessica Zucman-Rossi (Inserm U674/1162, Paris).

- Nonvoting members:

Marianne L'hennaff (ARCAT-TRT-5-CHV, France), Michèle Sizorn (SOS hépatites, France); one representative of INSERM-ANRS Pharmacovigilance team, Paris, France (Imane Amri, Alpha

Diallo), Mélanie Simony, Carole Cagnot (INSERM-ANRS, Paris, France), one member of Inserm Transfert, Paris, France (Alice Bousselet, Mireille Caralp, Jean-Marc Lacombe), and one representative of each pharmaceutical company (MSD, Janssen, Gilead, Abbvie, BMS, Roche).
Sponsor: Imane Amri, Alpha Diallo, Carole Cagnot, Mélanie Simony (INSERM-ANRS, Paris, France), Ventzi Petrov-Sanchez (coordinator).
Methodology and Coordinating Centre: Loubna Ayour, Jaouad Benhida, Fabrice Carrat (coordinator), Frederic Chau, Céline Dorival, Audrey Gilibert, Isabelle Goderel, Victorien Grard, Warda Hadi, Georges Haour, Godwin Mawuvi, Léa Mba Mintsa, Grégory Pannetier, François Pinot, Muriel Sudres, François Téloulé (Sorbonne Université & INSERM U1136, Paris, France).

CIRRAL group:
Nathalie Ganne-Carrié[1,], Cendrine Chaffaut[2], Isabelle Archambeaud[3], Louis d'Alteroche[4], Frédéric Oberti[5], Dominique Roulot[6], Christophe Moreno[7], Alexandre Louvet[8], Thông Dao[9], Romain Moirand[10], Odile Goria[11], Eric Nguyen-Khac[12], Nicolas Carbonell[13], Jean-Charles Duclos-Vallée[14], Stanislas Pol[15], Victor de Ledinghen[16], Violaine Ozenne[17], Jean Henrion[18], Jean-Marie Péron[19], Albert Tran[20], Gabriel Perlemuter[21], Xavier Amiot[22], Jean-Pierre Zarski[23], Sylvie Chevret[2].
[1]AP-HP, Hôpital Avicenne, Service d'Hépatologie, Bobigny, Université Sorbonne Paris Nord, Bobigny et INSERM U1138, Université de Paris; [2]SBIM, APHP, Hôpital Saint-Louis, Paris, Inserm, UMR-1153, ECSTRA Team, Paris, France; [3]Liver, CHU, Nantes, France; [4]Liver Unit, University Hospital, Tours, France; [5]Liver Unit, University Hospital, Angers, France; [6]AP-HP, Hôpital Avicenne, Service de Médecine Interne, Bobigny, Université Sorbonne Paris Nord, Bobigny; [7]Liver unit, CUB Hôpital Erasme, Université Libre de Bruxelles, Belgium, [8]Liver Unit, University Hospital, Lille, France, [9]Liver Unit, University Hospital, Caen, France ; [10]Liver Unit, University Hospital, Rennes, France ; [11]Liver Unit, University Hospital, Rouen, France ; [12]Liver Unit, University Hospital, Amiens, France ; [13] Liver Unit, APHP, CHU Saint-Antoine, Paris, France ; [14]Liver Unit, APHP, CHU Paul Brousse, Villejuif, France; [15]Université Paris Descartes; APHP, Liver Unit, Hôpital Cochin; INSERM U1223, Institut Pasteur, Paris, France; [16]Hepatology Unit, University Hospital, CHU Bordeaux, France; [17]Liver Unit, APHP, CHU Lariboisière, Paris, France; [18]Liver Unit, University Hospital, Haine Saint-Paul, Belgium, [19]Liver Unit, Universitary Hospital Purpan, University Paul Sabatier III, Toulouse ; [20]Institut National de la Santé et de la Recherche Médicale (INSERM), U1065, Team 8, "Hepatic Complications in Obesity", Nice, F-06204, Cedex 3, France, University Hospital of Nice, Digestive Centre, Nice, F-06202, Cedex 3, France ; [231]Liver Unit, University Hospital, Béclère, APHP, Clamart, France; [22]Liver Unit, APHP, CHU Tenon, Paris, France; [23]Clinique d'hépato-gastroentérologie pôle Digidune CHU de Grenoble, France
Funding/Support: The promoter of the study was the Assistance Publique des Hôpitaux de Paris (APHP), and the cohort was funded by the Institut National du Cancer (INCa).
Role of the Sponsor: The funding sponsor had no role in the design and conduct of the study; collection, management, analysis, interpretation of the data, and preparation, review, or approval of the manuscript.

Supplementary figures

Fig. S1. Constitution of the training and validation sets. Centers from the CirVir ($A_1 \ldots A_l$), Cirral ($B_1 \ldots B_m$), and Hepather ($C_1 \ldots C_n$) cohorts were pooled to create the total study population. This population was then split into a training set (approximately two-thirds of patients) and a validation set (the remaining one-third). To preserve center-level independence, all patients from a given center were randomly assigned together to either the training or the validation set.

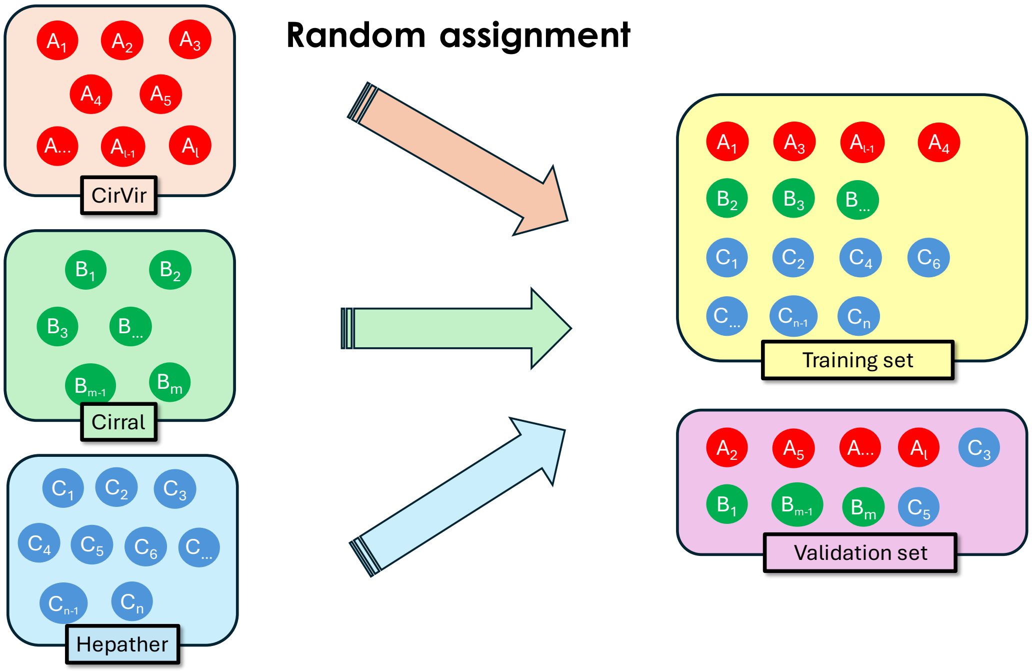

Fig. S2. Out of bag error rate against number of trees. A minimal number of 1000 trees is required to reach stability of the Out-Of-Bag error rate.

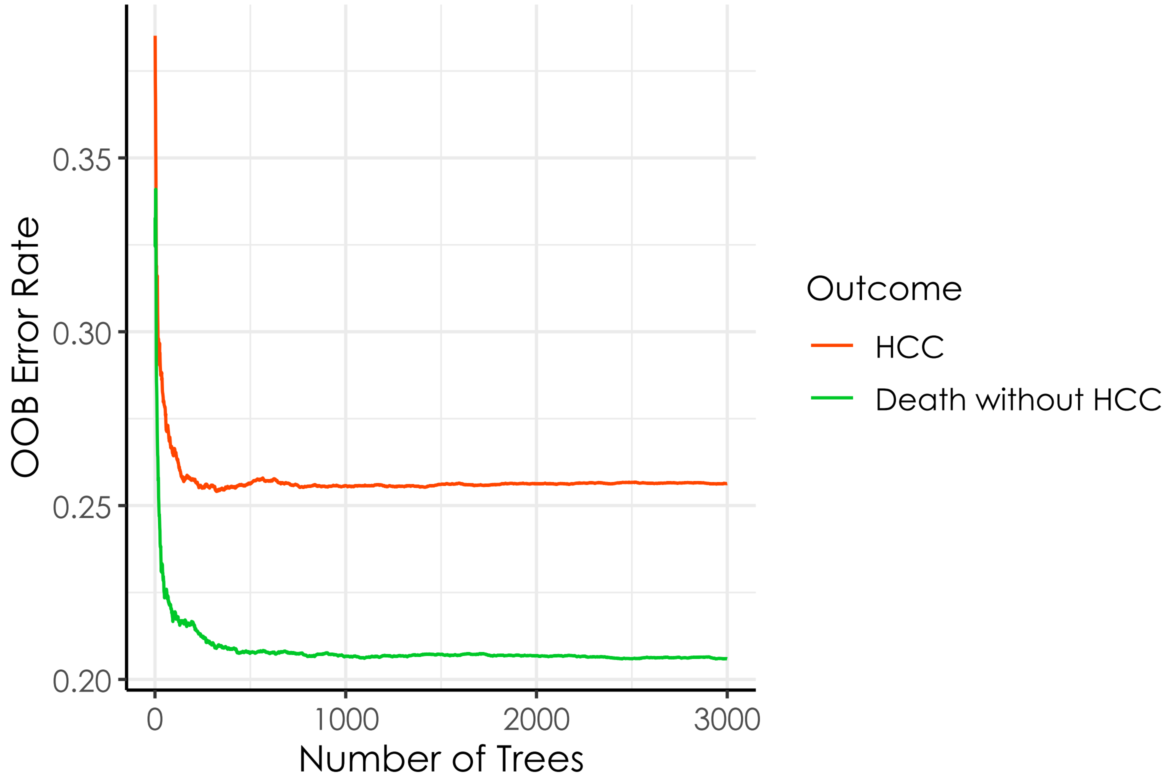

Fig. S3. Flowchart

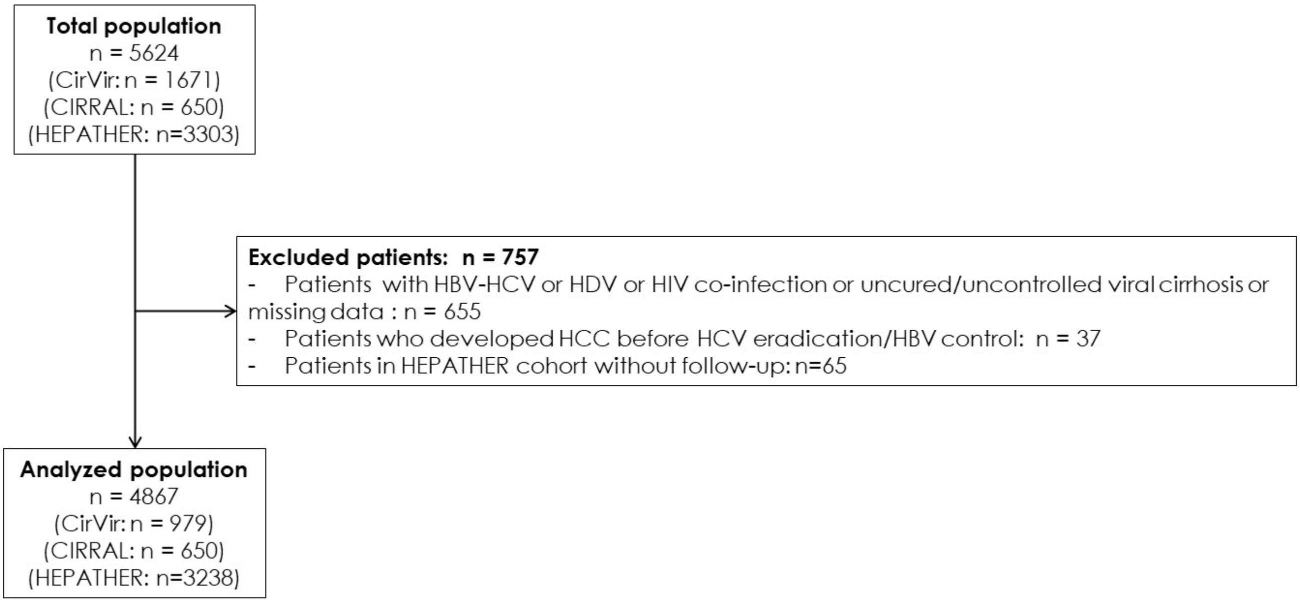

Fig. S4. HCC incidence in the development (n=3251) and validation (n=1616) cohorts. Levels of significance: P<0.05 (*Gray's test)*

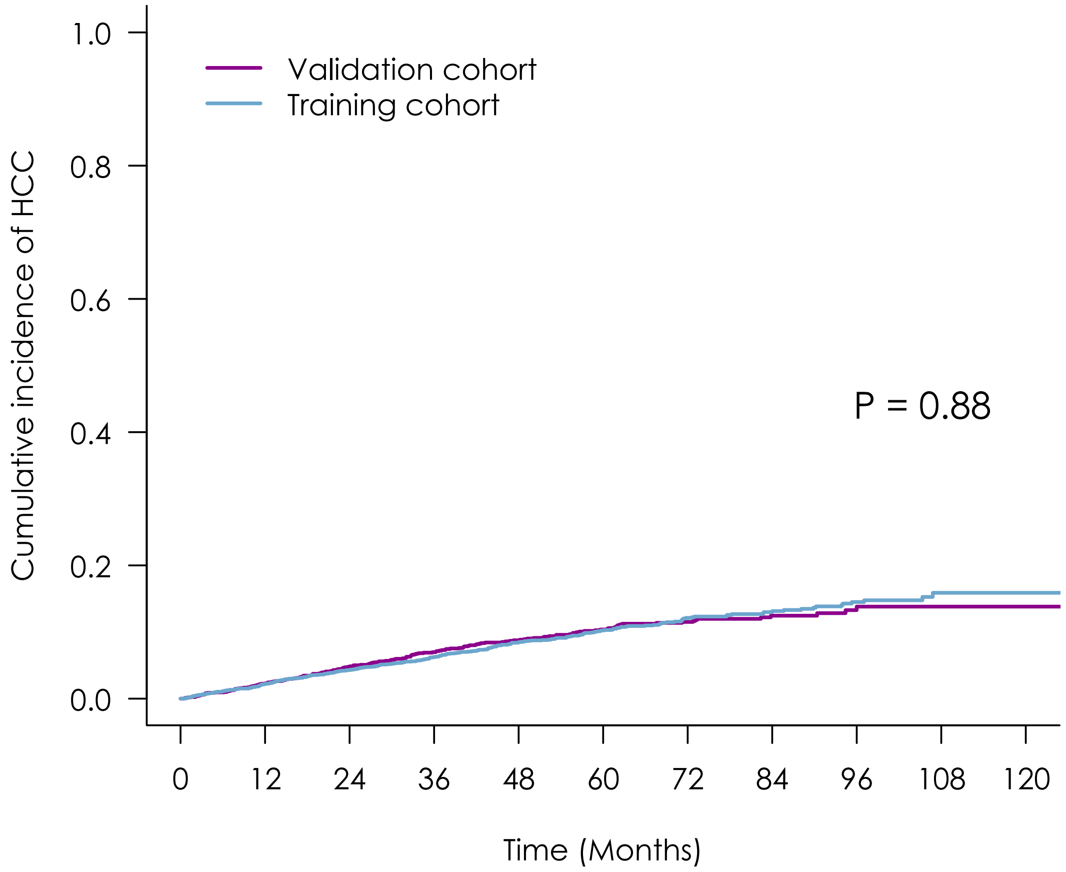

Supplementary tables

Table S1. Baseline characteristics of each cohort

Characteristic	N	Overall N = 4867[1]	CIRRAL N=650[1]	CIRVIR N=979[1]	HEPATHER N=3238[1]	p-value[2]
Age	4867	58.1 [52.0;66.0]	58.0 [51.0;64.0]	57.0 [50.0;65.0]	58.8 [52.5;66.9]	**<0.001**
Male sex		3,280 (67.4%)	438 (67.4)	669 (68.3)	2,173 (67.1)	
Platelet count, $10^3/mm^3$	4223	160 [113;208]	145 [104;191]	159 [110;202]	165 [117;213]	**<0.001**
AST	4259	29.0 [23.0;39.0]	33.0 [26.0;49.0]	31.0 [25.0;41.0]	28.0 [22.0;36.5]	**<0.001**
ALT	4397	26.0 [19.0;38.0]	26.0 [20.0;39.0]	29.0 [21.0;43.0]	25.0 [19.0;36.0]	**<0.001**
GGT	4082	45.0 [26.0;93.0]	108 [52.0;217]	42.0 [25.0;73.0]	39.0 [24.0;74.0]	**<0.001**
Prothrombin time, %	2955	87.0 [76.0;97.0]	78.0 [67.0;90.0]	89.0 [81.0;99.0]	88.0 [78.0;97.0]	**<0.001**
Serum albumin, g/L	3352	42.0 [38.9;45.0]	40.5 [37.0;43.2]	43.0 [40.0;46.0]	42.0 [39.0;45.0]	**<0.001**
Total bilirubin, µmol/L	3578	11.0 [8.0;16.1]	14.0 [10.0;20.0]	10.0 [7.0;14.0]	11.0 [8.0;16.0]	**<0.001**
Alpha-fetoprotein, ng/mL	3842	5.2 [3.00;10.4]	3.80 [2.50;5.5]	3.00 [2.00;5.0]	7.0 [3.80;13.7]	**<0.001**
INR	2685	1.10 [1.02;1.20]	1.20 [1.10;1.30]	1.10 [1.10;1.14]	1.10 [1.02;1.20]	**<0.001**
Creatinin	3139	71.0 [61.3;83.6]	70.0 [60.0;82.7]	74.0 [63.0;86.0]	70.7 [61.3;82.7]	**<0.001**
Glycemia	2851	1.03 [0.90;1.60]	5.6 [5.00;6.7]	0.99 [0.88;1.10]	0.99 [0.89;1.18]	**<0.001**
Alcalines phospatases	4019	88.0 [68.0;121]	96.0 [73.0;133]	83.5 [65.0;116]	88.8 [69.0;118]	**<0.001**
Ferritin	4867	233 [120;427]	112 [68.9;215]	234 [122;467]	260 [142;444]	**<0.001**
Cirrhosis aetiology	4867					
Cured HCV		3,543 (72.8%)	0 (0.0)	742 (75.8)	2,801 (86.5)	
Controlled HBV		674 (13.8%)	0 (0.0)	237 (24.2)	437 (13.5)	
Alcohol and/or Met-ALD		650 (13.4%)	650 (100.0)	0 (0.0)	0 (0.0)	

[1]Median [Q1;Q3]; n (%)

[2]Kruskal-Wallis rank sum test; Fisher's exact test. *Levels of significance: P<0.05*

Table S2. Discrimination performance indices by cohort (validation sets)

Time-dependent AUC	CirVir	CIRRAL	HEPATHER
**At 1 year**			
aMAP score	0.7641 (0.6528 ; 0.8755)	0.8505 (0.6493 ; 1.00)	0.6979 (0.5960 ; 0.7998)
FASTRAK score	0.7127 (0.5229 ; 0.8995)	0.8262 (0.6336 ; 1.00)	0.7136 (0.6146 ; 0.8125)
Decision tree	0.6066 (0.3956 ; 0.8176)	0.5815 (0.3435 ; 0.8195)	0.7110 (0.6046 ; 0.8174)
Survival random forest	0.7393 (0.5815 ; 0.8971)	0.6809 (0.4866 ; 0.8752)	0.7594 (0.6647 ; 0.8541)
Deep neural network	0.7694 (0.6605 ; 0.8783)	0.7544 (0.5826 ; 0.9261)	0.7534 (0.6481 ; 0.8583)
**At 3 years**			
aMAP score	0.6782 (0.5163 ; 0.8401)	0.9198 (0.8618 ; 0.9777)	0.6475 (0.5805 ; 0.7145)
FASTRAK score	0.6735 (0.5475 ; 0.7995)	0.8469 (0.7741 ; 0.9197)	0.7007 (0.6366 ; 0.7648)
Decision tree	0.6073 (0.4740 ; 0.7406)	0.6748 (0.5373 ; 0.8123)	0.6785 (0.6143 ; 0.7426)
Survival random forest	0.6748 (0.5667 ; 0.7829)	0.7783 (0.6951 ; 0.8616)	0.7281 (0.6670 ; 0.7891)
Deep neural network	0.7427 (0.6492 ; 0.8362)	0.8623 (0.8010 ; 0.9235)	0.6996 (0.6323 ; 0.7670)
**At 5 years**			
aMAP score	0.7326 (0.5994 ; 0.8659)	0.8523 (0.7293 ; 0.9753)	0.6234 (0.5667 ; 0.6800)
FASTRAK score	0.7199 (0.6190 ; 0.8209)	0.8201 (0.7359 ; 0.9043)	0.6558 (0.5962 ; 0.7155)
Decision tree	0.6246 (0.5019 ; 0.7474)	0.6558 (0.5341 ; 0.7775)	0.6483 (0.5923 ; 0.7042)
Survival random forest	0.7084 (0.6145 ; 0.8022)	0.7363 (0.6453 ; 0.8273)	0.6733 (0.6123 ; 0.7343)
Deep neural network	0.7973 (0.7109 ; 0.8837)	0.7543 (0.6402 ; 0.8684)	0.6669 (0.6049 ; 0.7289)

Brier score	CirVir	CIRRAL	HEPATHER
**At 1 year**			
aMAP score	0.019 (0.004 ; 0.034)	0.016 (0.001 ; 0.031)	0.023 (0.015 ; 0.032)
FASTRAK score	0.019 (0.004 ; 0.034)	0.016 (0.001 ; 0.061)	0.023 (0.015 ; 0.032)
Decision tree	0.019 (0.005 ; 0.034)	0.018 (0.002 ; 0.033)	0.023 (0.014 ; 0.032)
Survival random forest	0.019 (0.004 ; 0.035)	0.017 (0.002 ; 0.033)	0.023 (0.014 ; 0.032)
Deep neural network	0.019 (0.005 ; 0.034)	0.018 (0.004 ; 0.033)	0.023 (0.015 ; 0.031)
**At 3 years**			
aMAP score	0.052 (0.028 ; 0.076)	0.058 (0.032 ; 0.084)	0.065 (0.051 ; 0.078)
FASTRAK score	0.052 (0.028 ; 0.076)	0.060 (0.034 ; 0.085)	0.063 (0.050 ; 0.076)
Decision tree	0.052 (0.028 ; 0.077)	0.065 (0.039 ; 0.092)	0.063 (0.050 ; 0.077)
Survival random forest	0.051 (0.027 ; 0.074)	0.066 (0.038 ; 0.093)	0.063 (0.049 ; 0.076)
Deep neural network	0.052 (0.030 ; 0.074)	0.063 (0.039 ; 0.086)	0.063 (0.051 ; 0.075)
**At 5 years**			
aMAP score	0.072 (0.044 ; 0.100)	0.084 (0.052 ; 0.115)	0.092 (0.077 ; 0.107)
FASTRAK score	0.073 (0.044 ; 0.102)	0.087 (0.056 ; 0.117)	0.089 (0.074 ; 0.103)
Decision tree	0.076 (0.048 ; 0.104)	0.096 (0.066 ; 0.125)	0.089 (0.074 ; 0.103)
Survival random forest	0.073 (0.046 ; 0.101)	0.094 (0.063 ; 0.125)	0.088 (0.073 ; 0.102)
Deep neural network	0.075 (0.052 ; 0.098)	0.097 (0.071 ; 0.124)	0.092 (0.079 ; 0.104)
